# Supplementary material for: Differentiation of subnucleus-sized oligomers and nucleation-competent assemblies of the Aβ peptide
Source: Biophys J. 2022 Dec 17;122(2):269–78. doi: 10.1016/j.bpj.2022.12.020 (PMC9892607; doi:10.1016/j.bpj.2022.12.020)
Supplement: Document S1. Supporting materials and methods and Figures S1–S8 [file mmc1.pdf]

**Biophysical Journal, Volume *122***

## Supplemental information

# Differentiation of subnucleus-sized oligomers and nucleation-competent assemblies of the A$\beta$ peptide

**Thomas Pauly, Tao Zhang, Rebecca Sternke-Hoffmann, Luitgard Nagel-Steger, and Dieter Willbold**

Manuscript submitted to **Biophysical** *Journal*

**Article**

# Differentiation of sub-nucleus sized oligomers and nucleation competent assemblies of the Aβ peptide

Thomas Pauly[1,2], Tao Zhang[1,2,3], Rebecca Sternke-Hoffmann[1,4], Luitgard Nagel-Steger[1,2,*], and Dieter Willbold[1,2]

[1]Institut für Physikalische Biologie, Heinrich-Heine-Universität Düsseldorf, 40225 Düsseldorf, Germany
[2]Institute of Biological Information Processing (IBI-7: Structural Biochemistry), Research Center Jülich, 52425 Jülich, Germany
[3]Present address: Fujian Key Laboratory of Translational Research in Cancer and Neurodegenerative Diseases, Institute for Translational Medicine, School of Basic Medical Sciences, Fujian Medical University, Fuzhou, Fujian 350122, China.
[4]Present address: Department of Biology and Chemistry, OFLC/108, Paul Scherrer Institut, Forschungsstrasse 111, 5232 Villingen, Switzerland
[*]Correspondence: luitgard.nagel-steger@hhu.de

## SUPPLEMENTARY MATERIAL

### Material and methods

### Analytical ultracentrifugation

Due to the applied centrifugal forces hydrostatic pressure is build up in the sample forming a gradient with a maximum at the bottom of the cell. Since changing pressure can affect macromolecular structure or chemical equilibria, it is helpful to calculate with 1 how high this pressure is under the selected experimental conditions.

$$P = \omega^2 \rho (r_0 h + h^2/2) \tag{1}$$

In 1 $P$ is the hydrostatic pressure above atmospheric pressure, $\omega$ the angular velocity, $\rho$ the density of the fluid column, $r_0$ the radial distance from the axis of rotation to the meniscus , and $h$ the radial position in the cell: the liquid column height (1).

In order to test for the impact of these side effects of centrifugation either the solution column can be reduced or the speed can be lowered. A run with reduced speed was chosen resulting not only in reduced hydrostatic pressure but also in less shear forces and less pronounced solvent inhomogeneity. The difference in hydrostatic pressure at the bottom of the cell by changing the speed from 60 kprm ( 294 bar at 7.19 cm) und 50 kprm (202 bar at 7.18 cm) is about 90 bar.

### ThT data

The obtained ThT fluorescence data were fitted by a sigmoidal function that allows to obtain the lag time of amyloid fibrils formation (2). The fitted parameters are the elongation rate constant ($k$), the half completion time ($t^{1/2}$), the lower fluorescence baseline ($F_0$), and the amplitude to reach the upper baseline ($A$).

$$F(t) = F_0 + \frac{A}{1 + \exp(-k(t - t^{1/2}))} \tag{2}$$

### AFM imaging

Aβ42 in 20 mM Sodium phosphate buffer with 25 mM sodium chloride was incubated for 48 h before dilution from 40 to 20 μM and preparation of AFM samples. MICA surfaces were incubated for 20 minutes with 10 μL peptide solution, followed by washing steps with water and dried with nitrogen. Images were obtained using a NanoScope V (Bruker) atomic force microscope equipped with a silicon cantilever ScanAsyst-Air with a tip radius of 2-12 nm. Images were processed with Gwyddion 2.56 for presentation.

## Fluorescence dyes

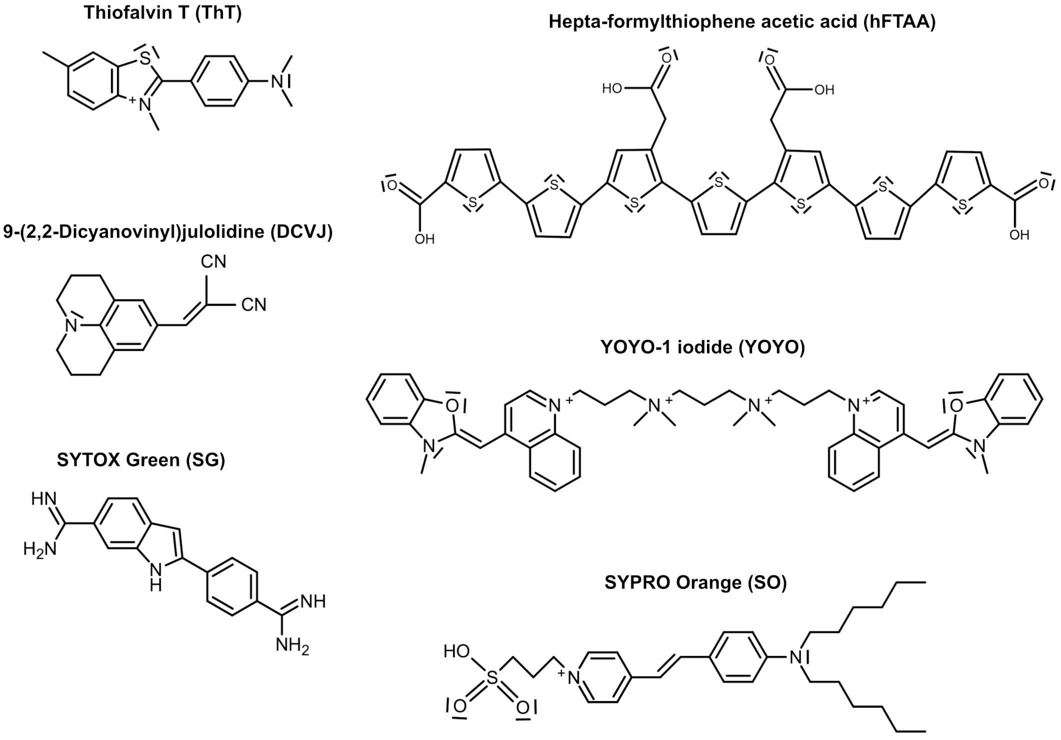


**Figure S1:** Molecular structures of fluorescent dyes used in this study.

**Table S1:** Fluorescent dyes with respective net charge and solvent used in this study

| **Fluorescence dye** | **Source** | **Solvent** | **Charge** [$\times 1.602\times 10^{-19}$ C] |
|---|---|---|---|
| ThT | Sigma-Aldrich, Germany | Water | + 1 |
| DCVJ | Sigma-Aldrich, Germany | DMSO | neutral |
| SO | Sigma-Aldrich, Germany | DMSO | zwitterionic |
| hFTAA | Nilsson and Hammarström (3) | Water | − 4 |
| YOYO | ThermoFisher, Germany | DMSO | + 4 |

## Results

**Table S2:** Weighted average *s*-value for oligomers from *c*(*s*) distributions.

| Sample | Detection type | average *s*-value [S] |
|---|---|---|
| | | **Oligomers from 4 to 20 S** |
| Aβ42 | Absorbance | 9.16 |
| Aβ42 + SO | Fluorescence | 8.63 |
| Aβ42 + hFTAA | Fluorescence | 8.66 |
| Aβ42 + SG | Fluorescence | 9.03 |
| Aβ42 + YOYO | Fluorescence | 11.02 |
| Aβ42 + DCVJ | Fluorescence | 9.24 |
| | | **Oligomers from 2 to 20 S** |
| Aβ42 | Absorbance | 8.65 |
| Aβ42 M35$^{ox}$ | Absorbance | 6.92 |
| Aβ40 | Absorbance | 7.12 |

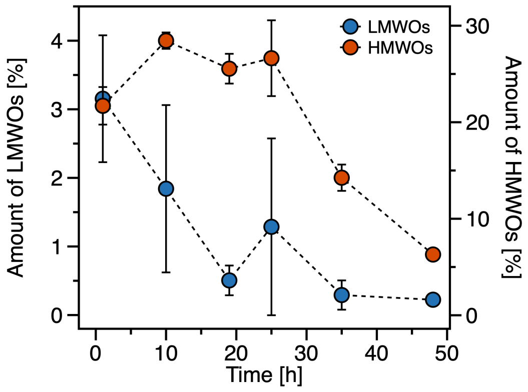


**Figure S2:** Amount of small oligomers and clusters of 40 μM Aβ42 for different incubation time points as the result of peak integration from 1.2 S to 4 S (blue dots) and 4 S to 20 S (red dots), respectively.

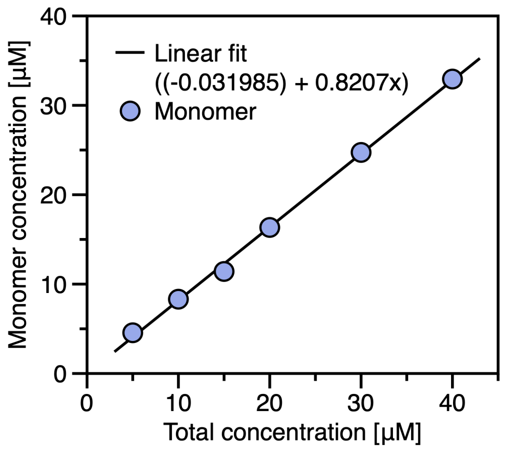


**Figure S3:** Integration of monomer peak from *c*(*s*) distributions obtained for a concentration series of Aβ42 without pre-incubation in 20 mM NaPi with 25 mM NaCl (pH 7.4). The monomer concentration dependence from total peptide concentration can be described by a linear relation between 5 and 40 μM.

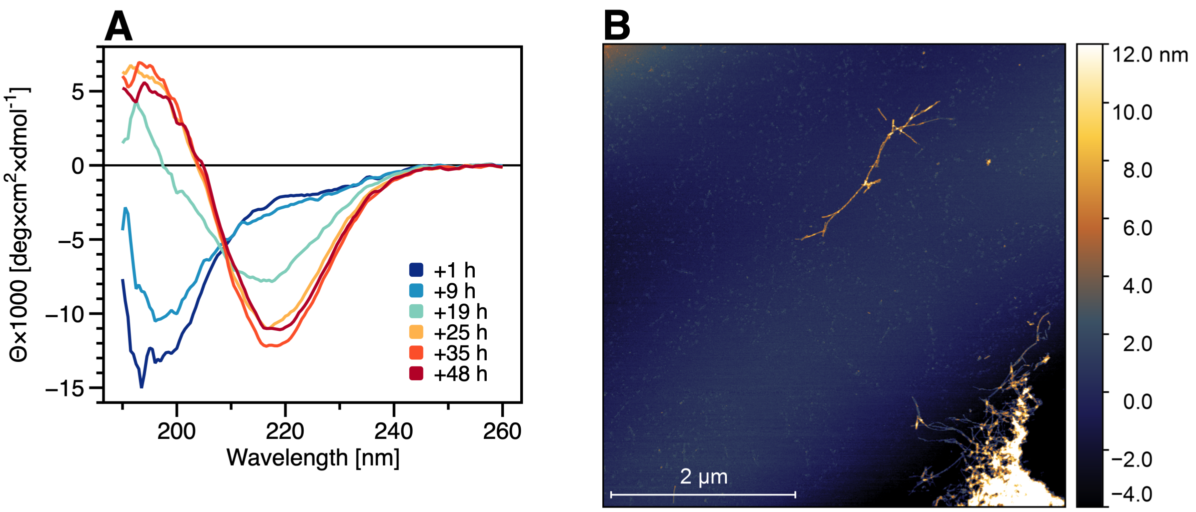


**Figure S4:** A shows CD spectra for 40 µM Aβ42 after different incubation times. B shows an AFM image of 20 µM Aβ42 after 48 h incubation. Samples were taken from kinetic experiments with 40 µM Aβ42 and diluted with 20 mM NaPi and 25 mM NaCl (pH 7.4).

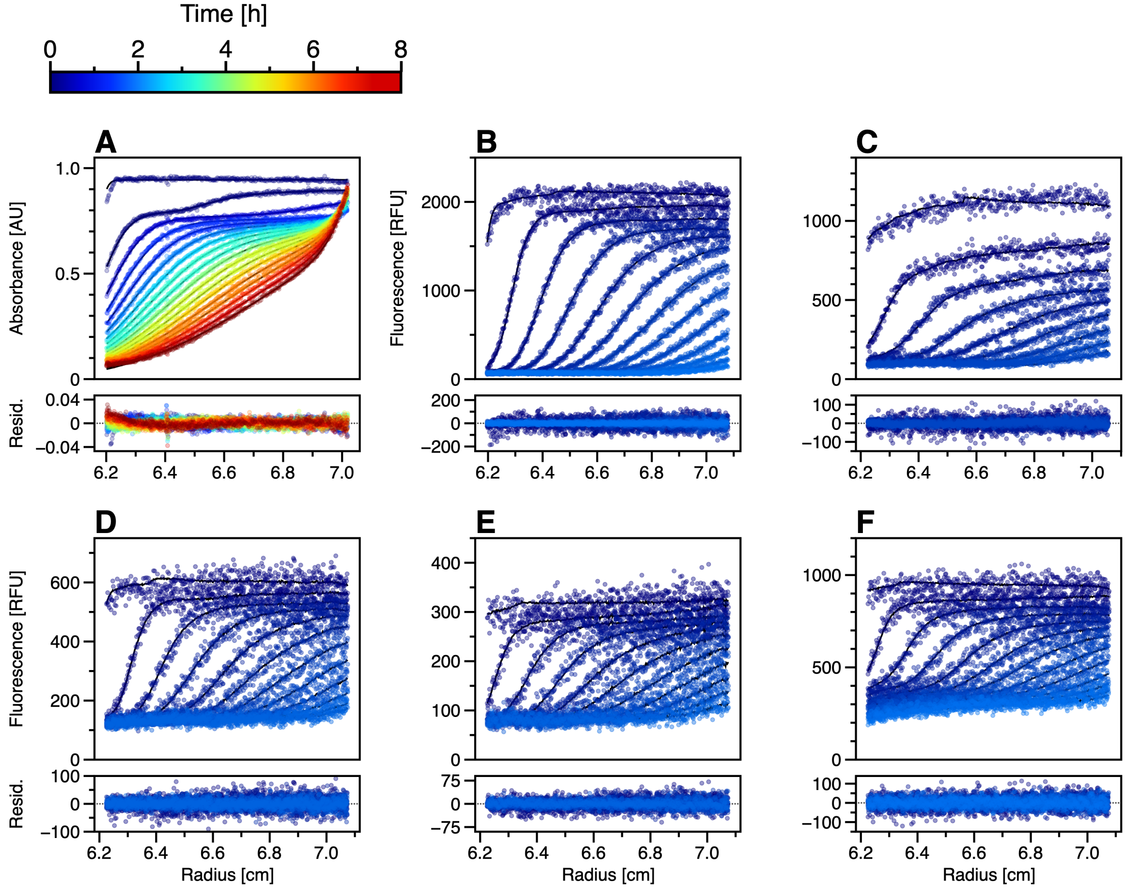


**Figure S5:** Sedimentation profiles of 40 µM Aβ42 after 6 h incubation measured with absorbance detection (A) and with fluorescence detection in the presence of different dyes: SO (B), YOYO (C), SYTOX (D), DCVJ (E) and hFTAA (F). The raw data is shown as points with fitted Lamm-equations solutions from the c(s) model implemented in Sedfit. For clarity, only every seventh scan is shown.

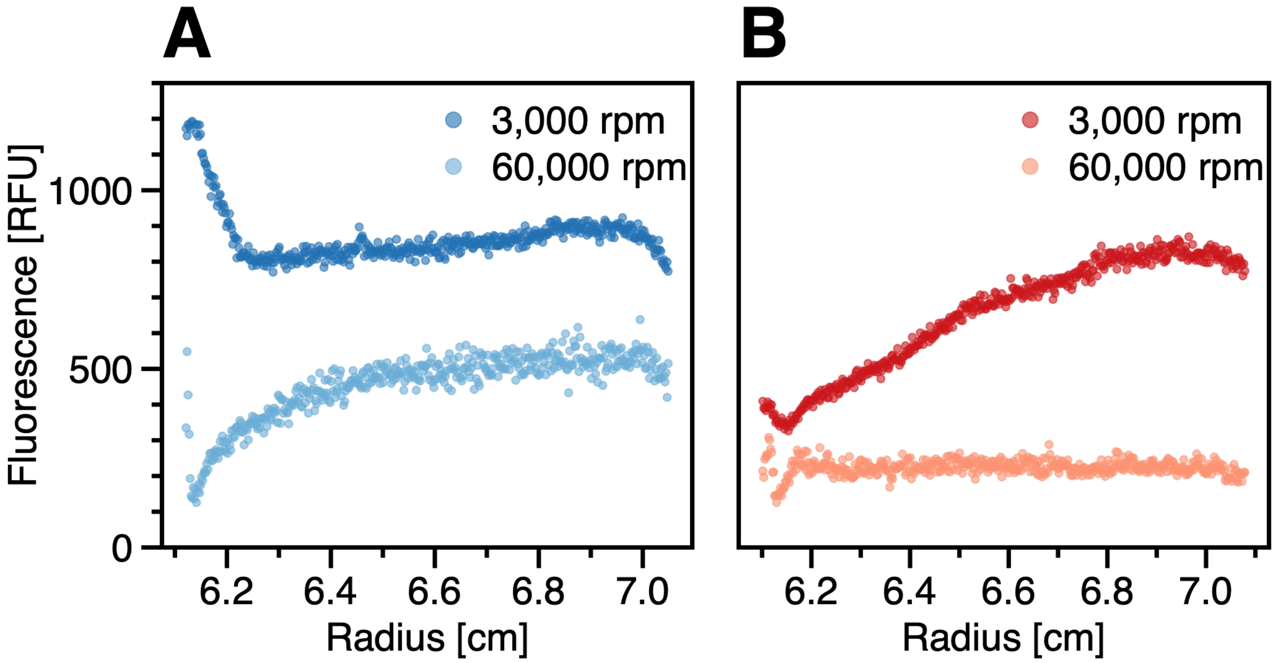


**Figure S6:** Loss of signal during incubation of 40 µM Aβ42 in the presence of YOYO. A single scan at 3,000 rpm and 60,000 rpm is shown for incubation of Aβ42 in the presence of YOYO for 1 h (A) and 6 h (B). The total incubation time of Aβ42 was 6 h for both samples.

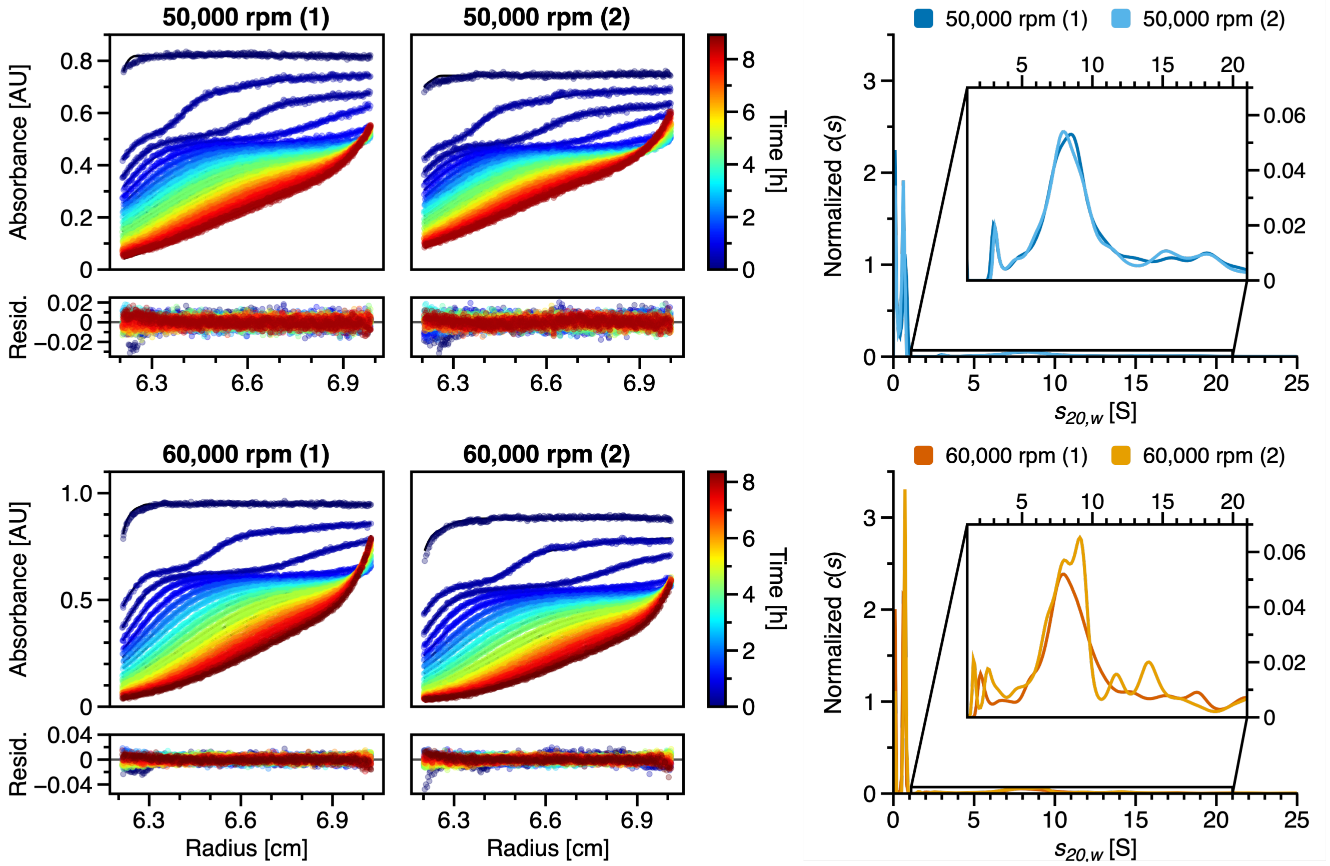


**Figure S7:** Sedimentation velocity analysis of 40 μM Aβ42 after 3 h incubation at 50,000 rpm and 60,000 rpm. Sedimentation profiles of duplicates show the raw data as points with fitted Lamm-equation solutions from the $c(s)$ model implemented in Sedfit. The resulting distribution of $s$-values is shown for duplicates of both speeds with magnification of oligomer distributions between 1 S and 21 S. Curves are normalized for the complete area under curve.

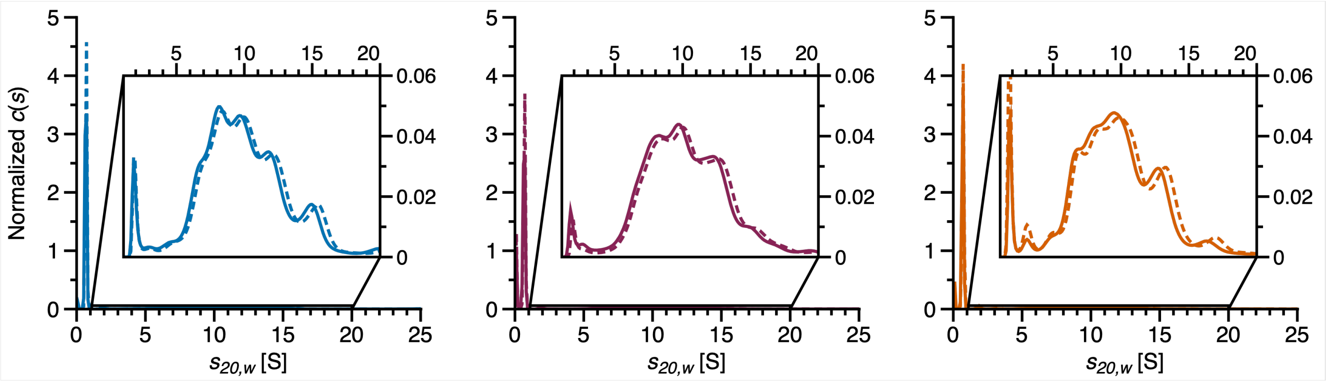


**Figure S8:** Comparison of sedimentation velocity analyses of 40 μM Aβ42 after 10 h incubation accounting for solvent compressibility. The data shown for triplicats from the main text (solid lines) were analyzed again using the $c(s)$ model with solvent compressibility (dashed lines) implemented in Sedfit.

## REFERENCES

1. Molina-García, A., 1999. Hydrostatic pressure in ultracentrifugation. *In* Progress in Colloid and Polymer Science. Springer Verlag, volume 113, 57–61.

2. Hellstrand, E., B. Boland, D. M. Walsh, and S. Linse, 2010. Amyloid $\beta$-protein aggregation produces highly reproducible kinetic data and occurs by a two-phase process. *ACS chemical neuroscience* 1:13–18.

3. Klingstedt, T., A. Åslund, R. A. Simon, L. B. Johansson, J. J. Mason, S. Nyström, P. Hammarström, and K. P. R. Nilsson, 2011. Synthesis of a library of oligothiophenes and their utilization as fluorescent ligands for spectral assignment of protein aggregates. *Organic & biomolecular chemistry* 9:8356–8370.
