# Supplementary material for: Differentiation of subnucleus-sized oligomers and nucleation-competent assemblies of the Aβ peptide
Source: Biophys J. 2022 Dec 17;122(2):269–78. doi: 10.1016/j.bpj.2022.12.020 (PMC9892607; doi:10.1016/j.bpj.2022.12.020)
Supplement: Document S2. Article plus supporting material [file mmc2.pdf]

Biophysical Journal
Article

Biophysical Society

Differentiation of subnucleus-sized oligomers and nucleation-competent assemblies of the Aβ peptide

Thomas Pauly,[1,2] Tao Zhang,[1,2] Rebecca Sternke-Hoffmann,[1] Luitgard Nagel-Steger,[1,2,*] and Dieter Willbold[1,2]
[1]Institut für Physikalische Biologie, Heinrich-Heine-Universität Düsseldorf, Düsseldorf, Germany and [2]Institute of Biological Information Processing (IBI-7: Structural Biochemistry), Research Center Jülich, Jülich, Germany

ABSTRACT A significant feature of Alzheimer's disease is the formation of amyloid deposits in the brain consisting mainly of misfolded derivatives of proteolytic cleavage products of the amyloid precursor protein amyloid-β (Aβ) peptide. While high-resolution structures already exist for both the monomer and the amyloid fibril of the Aβ peptide, the mechanism of amyloid formation itself still defies precise characterization. In this study, low and high molecular weight oligomers (LMWOs and HMWOs) were identified by sedimentation velocity analysis, and for the first time, the temporal evolution of oligomer size distributions was correlated with the kinetics of amyloid formation as determined by thioflavin T-binding studies. LMWOs of subnucleus size contain fewer than seven monomer units and exist alongside a heterogeneous group of HMWOs with 20–160 monomer units that represent potential centers of nucleus formation due to high local monomer concentrations. These HMWOs already have slightly increased β-strand content and appear structurally similar regardless of size, as shown by examination with a range of fluorescent dyes. Once fibril nuclei are formed, the monomer concentration begins to decrease, followed by a decrease in oligomer concentration, starting with LMWOs, which are the least stable species. The observed behavior classifies the two LMWOs as off pathway. In contrast, we consider HMWOs to be on-pathway, prefibrillar intermediates, representing structures in which nucleated conformational conversion is facilitated by high local concentrations. Aβ40 and Aβ42 M35^{ox} take much longer to form nuclei and enter the growth phase than Aβ42 under identical reaction conditions, presumably because both the size and the concentration of HMWOs formed are much smaller.

SIGNIFICANCE Understanding the molecular mechanisms of how prefibrillar intermediates convert into amyloid fibrils is critical for exploring therapeutic options to combat amyloid-related diseases. Sedimentation velocity experiments show the coexistence of at least three different types of oligomeric Aβ42 species. Small oligomers (LMWOs) disappear before larger oligomers (HMWOs) when the reaction enters the growth phase. HMWOs constitute potential nucleation sites by providing high local monomer concentrations facilitating nucleus formation. Lower formation and smaller size of HMWOs from Aβ40 and Aβ42 M35^{ox} have been shown to be associated with lower fibril formation compared with Aβ42.

INTRODUCTION

In their recent review about the amyloid β (Aβ) oligomer hypothesis, Cline et al. (1) reported that an increasing number of publications support the idea that toxic oligomeric species of the Aβ peptide (AβO) are key players in Alzheimer's disease (AD). This switch in focus is already reflected in an increasing number of AβO-targeting therapeutics in the pipeline aiming for a breakthrough in AD therapy (2,3). Nevertheless, it is still not clear what type of oligomeric species among the multitude of described Aβ assemblies is a promising target with regard to its size and structure. According to a recent survey, Aβ oligomers correlate poorly with preclinical Alzheimer's neurodegeneration (4). Therefore, it is essential to collect as much information about the nature of these AβOs as possible. Not only their structure but also the kinetics of their formation as well as their function within the framework of amyloid formation have to be resolved (1). In literature, a multitude of different oligomeric species of Aβ has been described regarding their size, structure, or function (5), e.g., toxic

Submitted July 29, 2022, and accepted for publication December 14, 2022.

*Correspondence: luitgard.nagel-steger@hhu.de

Tao Zhang's present address is Fujian Key Laboratory of Translational Research in Cancer and Neurodegenerative Diseases, Institute for Translational Medicine, School of Basic Medical Sciences, Fujian Medical University, Fuzhou, Fujian, China.

Rebecca Sternke-Hoffmann's present address is Department of Biology and Chemistry, OFLC/108, Paul Scherrer Institut, Villingen, Switzerland.

Editor: John Correia.

https://doi.org/10.1016/j.bpj.2022.12.020

© 2022 Biophysical Society.

This is an open access article under the CC BY license (http://creativecommons.org/licenses/by/4.0/).

effects are attributed to AβOs termed Aβ-derived diffusible ligands (ADDLs) (6) or to a 60-kDa oligomer termed Aβ42 globulomer, which has been detected in human brain (7). Additionally, it is possible that oligomeric species with different toxic properties exist, and that they play different roles in the pathology of AD (1,8). While fibril structures could be successfully solved for Aβ (9) and other amyloidogenic proteins (10) with high resolution, i.e., by combining solid-state NMR and cryo-electron microscopy, the structure of smaller assemblies of the involved proteins still resists a detailed characterization in atomic resolution (11). Analyzing the oligomeric species suffers from several obstacles like small quantities in relation to the total concentration of the protein, heterogeneous populations, and a very dynamic and transient nature, which need to be overcome. Referring to the classical nucleation theory, we apply the term nucleus for a metastable intermediate between monomer and fibril with the highest free energy (12). Such a nucleus is an assembly, which has a higher probability to grow than to dissociate (13). Further, we use the terms low molecular weight oligomers (LMWOs) and high molecular weight oligomers (HMWOs) to subdivide soluble Aβ oligomers according to their molecular mass. LMWOs by our definition are built out of less than seven monomeric units, while HMWOs include everything larger, e.g., dodecamers (14). The LMWOs and HMWOs mentioned in this report are Aβ42 intermediate species, which appear after dissolving Aβ42 and disappear in favor of fibrillar structures at the end of the incubation time. Sedimentation velocity (SV) centrifugation experiments were applied to characterize a group of reproducibly detectable s-value species as evaluated by $c(s)$ distribution analysis (15). The classified s-value species appear consistently independent of centrifugal speeds for different protein pretreatments and total protein concentrations. They can be detected over a wide range of incubation times, although their individual fractions vary with time. The majority of species can be found between 4 and 15 S. A small assembly with about 2.8 S, which is detectable at rather low amounts, has been characterized as penta-to hexamer of Aβ42 already (16). Here, analytical ultracentrifugation (AUC) with either absorbance or fluorescence detection in combination with thioflavin T-based kinetic measurements were used to analyze the evolution of Aβ42 oligomer compositions over time. SV centrifugation allows for the determination of size and shape distributions of macromolecular solutions. It is an absolute, matrix-free, solution-based method. In contrast to band centrifugation, the faster sedimenting species are not physically separated from the slower sedimenting components. This property of boundary centrifugation is very advantageous for the analysis of transient and dynamic assemblies as macromolecular complexes with high dissociation rates are thus protected from dissociation during the measurement (17). In addition, large fibrils or particles are quickly removed from solution due to their high sedimentation coefficient so that processes with fast kinetics such as fibril elongation and secondary nucleation do not interfere with the measurement. Although these processes are suppressed during SV centrifugation, they naturally occur during the incubation periods of the samples prior to AUC measurement and thus contribute to the results. Monitoring of molecular concentration gradients formed along the radius during exposure to high centrifugal fields, e.g., 260,000 $\times$ g (60,000 Rpm), was performed by either absorbance or fluorescence detection. The gravity field applied in AUC causes a hydrostatic pressure gradient along the radius of the sample cell, which can have a significant impact on a measurement. The presence of pressure-dependent reaction equilibria and density gradients could potentially alter the size distributions during the experiment. Although the method has some potential risks for the application described, the advantage of high resolution and sensitivity in characterizing the aggregates outweighs them, provided that adverse effects can be excluded by appropriate controls.

The use of the fluorescence detection system (FDS) in AUC enabled the study of Aβ aggregation at low concentrations with a covalently Alexa488-labeled Aβ42 monomer (16). The disadvantage of this method is the covalent labeling of the monomer unit, which can lead to changes in the aggregation pathway. In contrast, the use of an extrinsic fluorescent dye added to the sample immediately prior to analysis would minimize the potential effects of the dye on aggregation. This approach has been referred to as biological on-line tracer sedimentation (18). The use of fluorescent dyes is an established method for the detection of amyloid either in a tissue or in a solution. The two best known dyes are Congo red and thioflavin T (ThT) 11. Unfortunately, neither of these dyes is suitable for FDS of AUC, which offers only one excitation wavelength at 488 nm. Previous AUC measurements with Aβ42 incubated for 24 h and 9-(2,2-Dicyanovinyl)julolidine (DCVJ) (19,20) as an extrinsic fluorescence probe have shown promising results with staining oligomeric species between 2 and 20 S. SYPRO orange (SO) as a widely used protein stain can bind to amyloid fibrils (21,22) as well. In this study, we examined five different dyes that are suitable for the FDS in AUC and promising candidates for the detection of oligomeric species: DCVJ, SO, hepta-formylthiophene acetic acid (hFTAA) (23), SYTOX Green (SG), and YOYO-1 iodide (YOYO) (24) (see Fig. S1 for the chemical structures and Table S1 for the spectral properties of these dyes).

MATERIALS AND METHODS

All samples were prepared and incubated in Protein LoBind tubes from Eppendorf (Hamburg, Germany).

Buffers and solutions

Solutions were prepared using ultrapure water from a Milli-Q filtration system (Merck Millipore, Darmstadt, Germany). All experiments were

performed in 20 mM sodium phosphate buffer (pH 7.4) including 25 mM sodium chloride. Samples including fluorescent dyes contained 2% dimethyl sulfoxide of spectroscopic grade (Sigma-Aldrich, Darmstadt, Germany) as a vehicle to improve dissolution. Dimethyl sulfoxide was excluded from experiments with absorbance detection.

Aβ peptide and fluorophores

Aβ aliquots were prepared by dissolving 1 mg peptide (Bachem, Bubendorf, Switzerland, no. 4014447.1000) in 700 μL 1,1,1,3,3,3-hexafluoroisopropanol (Sigma-Aldrich) overnight at room temperature without agitation. Consequently, 1,1,1,3,3,3-hexafluoroisopropanol was removed in a vacuum concentrator. Dried peptide films were stored at -20°C until use. Any dissolution of such prepared Aβ42 aliquots started with the addition of less than 10% final volume of 10 mM NaOH before adjusting final solvent conditions. The quality of the peptide was examined by SV analysis of peptide dissolved in 10 mM NaOH, revealing $\geq$95% monomeric Aβ peptide (this experiment was performed in titanium cells because of their higher chemical resistance). Structures of the used fluorescence dyes are shown in Fig. S1, and a list of the dyes together with the solvent used to prepare the stock solutions is in Table S1.

AUC

SV experiments were performed in an AUC (Proteome Lab XL-A, Beckman-Coulter, Brea, CA, USA) at 60,000 Rpm, 20°C, and with a radial resolution of 0.002 cm in standard double-sector cells (optical path length: 1.2 cm) made of either aluminum (Beckman-Coulter) or titanium in case of extreme pH. At the beginning of each experiment, a wavelength scan at 3,000 Rpm was recorded to determine the optimal detection wavelength and used as a reference to quantify the amount of material sedimented during acceleration to 60,000 Rpm. The rotor used was an An-60Ti (4-hole) rotor from Beckman. The stated times of incubation were those times that passed between Aβ sample preparation and the start of sedimentation. SV data analysis was performed by applying a continuous distribution Lamm equation model, $c(s)$, as implemented in Sedfit (v.16p35) (25). Regularization was performed with maximum entropy algorithm, which favors the distribution with the lowest number of parameters (minimal information) to fit the data. Additionally, Datagraph (26) was used for data presentation. Solvent density and viscosity were calculated with Sednterp (27) (v.1.10, http://www.jphilo.mailway.com/sednterp.htm): $\rho = 1.0019$ g/cm^3 and $\eta = 0.01013$ Poise.

After incubation at room temperature, samples were loaded into 12-mm aluminum cells. The size distribution of Aβ42 was measured in triplicate with separate incubation for each time point, resulting in 21 independent aggregation processes, which were evaluated by SV analyses. If aggregation had taken place during incubation, we observed a loss of signal during acceleration of the centrifuge to final speed. This signal loss due to the sedimentation of large assemblies was accounted for in the final size distributions. The final $c(s)$ curves were transformed into standardized signal units representative of the sample concentration found when the final speed was achieved.

ThT kinetic measurement

For the ThT-based quantification of fibril mass, a 100-μL aliquot of the 40-μM Aβ42 sample for SV experiments was taken, and 1 μL ThT stock solution was added for a final concentration of 20 μM. Samples were measured in LoBind multiwell plates (Eppendorf, Hamburg, Germany) inside a microwell plate reader (Tecan, Männedorf, Switzerland). To prevent evaporation, plates were sealed. The ThT fluorescence was measured from the top with $\lambda_{ex} = 445$ nm and $\lambda_{em} = 485$ nm with a bandwidth of 9 nm each at 20°C without agitation.

CD spectroscopy

The secondary structure conversion of Aβ42 over time was characterized using circular dichroism (CD) spectroscopy. Aβ42 at 40 μM was prepared in 20 mM sodium phosphate and 25 mM sodium fluoride (pH 7.4). The exchange of chloride by fluoride increases the sensitivity of CD in the far UV region below 200 nm. Samples of 200 μL each were then loaded into a 1-mm quartz cuvette and were maintained at 20°C. CD spectra were recorded using a J-815 spectropolarimeter (Jasco, Tokyo, Japan) from 260 to 190 nm with a step size of 0.5 nm and a bandwidth of 2 nm. The scanning speed was 100 nm/min. For each time point, 10 scans were accumulated and averaged for a spectrum of the sample. Calculated mean residue ellipticities at either 216 or 198 nm were plotted against the incubation time to visualize the conversion kinetics.

RESULTS

In our study, we successfully merged kinetic information from ThT measurements with the corresponding aggregate size distributions measured by SV experiments. The essential requirement was a strictly controlled dissolution and incubation protocol for the Aβ peptide, which guaranteed high reproducibility for the aggregation process. To gain more detailed information on the intermediate species, several fluorescence dyes were tested for their suitability to stain Aβ42 LMWOs and HMWOs in SV experiments with fluorescence detection. Finally, we applied the advanced technique to variants of Aβ42, i.e. Aβ40 and Aβ42M35^{Ox}.

Aβ 42 ThT kinetics aligned with aggregate size distributions

ThT is a well-established fluorescent dye used as a marker for amyloid fibrils (28). It can be utilized in different types of assays either to quantify the amount of amyloid formed over time in a sample or to elucidate mechanistic properties of the process (reviewed in (29)). The ThT kinetic assay performed in parallel to the SV analyses allows the linkage of aggregation stages with s-value distributions for the underlying Aβ42 assemblies. Intermediate species between monomer and amyloid fibril are mostly invisible in ThT kinetics assay, probably due to the missing cross-β-sheet structure. SV analysis reports the s-value distribution of Aβ42 assemblies during the aggregation process, independent of the presence of cross-β structure.

SV analysis was performed in triplicate to analyze the distribution of s-values representing different species of Aβ42 at different stages of aggregation. The sedimentation profiles shown in Fig. 1 clearly demonstrate the high reproducibility of the aggregation process. The profiles already reveal, without further analysis, the existence of a faster-moving boundary resembling oligomeric species. These oligomeric species were sedimented within ~90 min during our experiments. These raw data were fitted to a model termed continuous distribution of sedimentation coefficients $c(s)$ (25), which is based on solutions of the Lamm equation (30). Integration of the obtained $c(s)$ distributions for two s-value

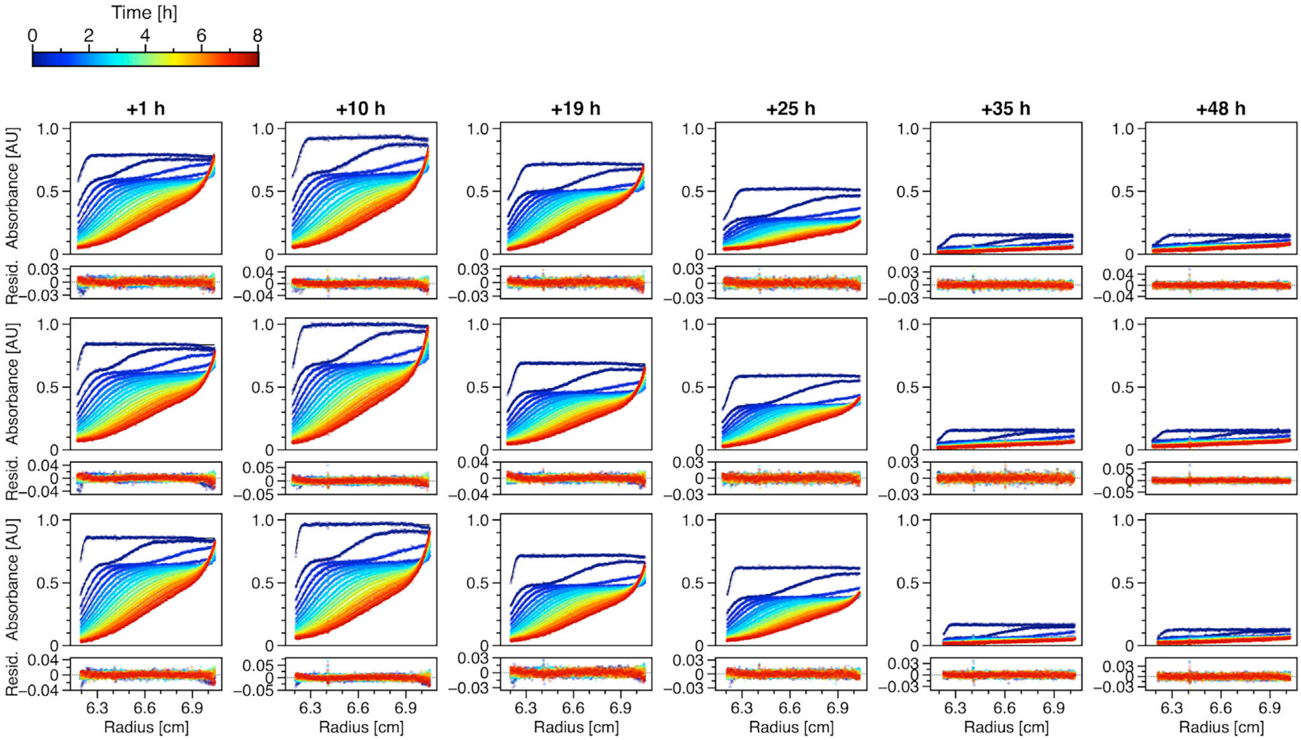

FIGURE 1 Sedimentation profiles of 40 μM Aβ42 after different incubation times (1, 10, 19, 25, 35, and 48 h) at 20°C in triplicates from top to bottom. The raw data (*colored dots*) are shown together with fitted Lamm equation solutions (*black lines*) from the $c(s)$ model implemented in Sedfit. For clarity, only every sixth scan is shown. Below each graph are the residuals depicted, i.e., the deviation of the fit from the measured data. Different colors indicate different time points of detection during sedimentation as shown in the legend on top. The detection wavelengths vary between different measurements. To see this figure in color, go online.

ranges, 0–1 S representing monomers and 1–20 S representing oligomers, yielded the corresponding fractions of monomers and oligomers over time (Fig. 2 *A*). The fraction of large aggregates was calculated for each experiment as a loss of signal during the acceleration of the centrifuge, and the corresponding averaged fractions are included in Fig. 2 *A*. The ThT fluorescence was measured for each sample directly before SV analysis (Fig. 2 *B*). To confirm the structural conversion of Aβ42, we additionally measured the CD signals for each time point (Fig. 2 *C*). For Aβ42, it is known that the soluble form is mostly unstructured and that amyloid formation is accompanied by increasing β-strand conformation (Fig. S4 *A*). Since CD is a bulk method, the measured spectra represent weight averages for all molecular species in solution independent of size or ThT positivity. The concomitant change in CD signal further supports the formation and growth of the amyloid structure during the incubation time. The small decrease of random-coil structure within the first 9 h of incubation may indicate fold reshuffling processes taking place in and among oligomeric species.

The combination of results from SV analyses and ThT fluorescence kinetics in Fig. 2, *A* and *B*, presents the evolution of species during the aggregation process. The time where the half maximum of the final ThT signal is reached is about 26 h for our system, and the duration of the lag phase is ∼18 h (calculated as the intercept of the time axis and the tangent from the midpoint of the curve (31)). The fraction of large aggregates was calculated based on the quantification of signal loss during acceleration due to aggregates, which are larger than approximately 208 S. It correlates well with the increase of ThT fluorescence and reaches a plateau at ∼87% of the total sample after 48 h. This correlation identifies these aggregates as ThT-positive amyloid structures. A negative correlation is found between the monomer fraction and ThT fluorescence. The class of oligomeric species reaches a maximum of ∼31% just before the entrance into the rapid growth phase after 10 h. The formation of oligomeric species appears to be fast, as they could already be detected after 1 h incubation. Their fraction shows a small increase in number for the first 10 h and a rather constant number until 24 h. The results also indicate that at least part of the intermediate species contribute to the ThT fluorescence signal, as we observe some fluorescence signal at the first two time points (1 and 20 h) before the entrance into the rapid growth phase. At 25 h, a strong decrease of the monomer fraction becomes evident, along with a sharp increase in the proportion of large aggregates, while the oligomeric fraction remains mostly unchanged. Only after the rapid depletion of monomers from the solution is a decrease in the oligomeric fraction observed.

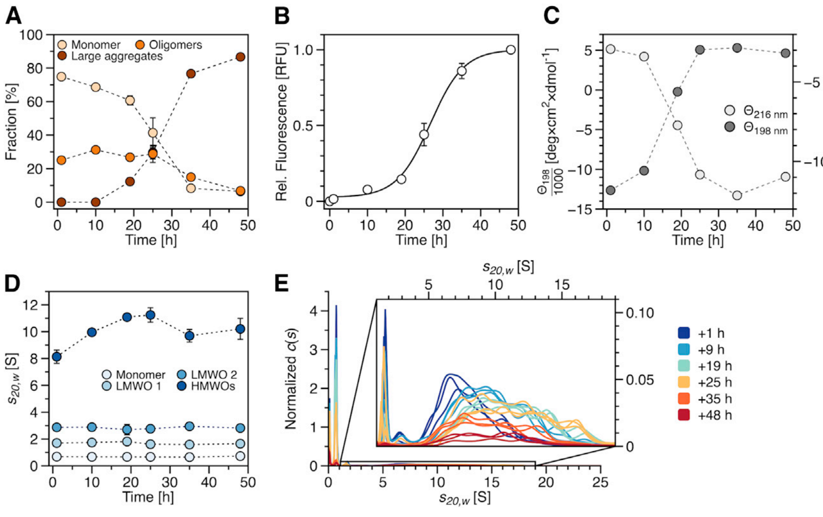

FIGURE 2 Amyloid formation kinetics for 40 μM Aβ42 in 20 mM sodium phosphate buffer (pH 7.4) with 25 mM NaCl or NaF, respectively, were tracked for six incubation time points with different techniques. (*A*) Time-dependent fraction of different *s*-value species determined by SV analyses. Each data point represents the fraction of the specific *s*-value species of the total amount of Aβ42 at a certain time point of incubation. The integrated signal from $c(s)$ distributions for a specific *s*-value range, transformed into percentage of total concentration, was used to calculate the fractions for monomers (0–1 S), oligomers (1–20 S), and large aggregates (>280 S) (*from yellow to red*). Data points represent mean values with standard deviations from triplicate measurements. Error bars may be hidden by dots. (*B*) ThT fluorescence measurements for triplicate samples over time representing amyloid fibril mass evolution; data (*white dots*) are fitted with Eq. 2 from (31) (*black line*). Error bars may be hidden by dots. (*C*) CD measurements over time. Mean residue ellipticity at 216 (*light gray dots*) and 198 nm (*dark gray dots*) with separate *y* axes. (*D*) Weight averaged *s*-values from peak integration for monomer and three classes of oligomers over time determined in triplicate (*from light to dark blue*). Error bars may be hidden by dots. (*E*) $c(s)$ distributions in triplicate for different incubation times. The magnification visualizes oligomeric species from 1 to 19 S. The $c(s)$ distributions were normalized to one, according to the area under the curve for the complete *s*-value range from 0 to maximum *s*-value and potential material loss, due to large aggregates sedimenting during acceleration was taken into account. Different colors indicate different incubation times. To see this figure in color, go online.

To visualize the amyloid fibrils at the end of the observed kinetic, images using atomic force microscopy (AFM) were taken from samples in the plateau of the ThT signal after 48 h (Fig. S4 *B*). The AFM images show fibrillar structures with height and diameter in agreement with amyloid fibrils both as single structures as well as superstructures like bundles or meshwork of fibrils.

SV analysis yields three distinct species of oligomers

Looking at the $c(s)$ distributions, it is evident that the oligomeric species belong to at least three distinct classes covering the *s*-value range from 1 to 20 S (Fig. 2 *E*). This *s*-value range translates into a molecular mass ranging from 10 to 800 kDa based on a frictional coefficient ratio (shape factor, f/f_0) of 1.5. This shape factor was calculated by using a variant of the $c(s)$ model with prior knowledge, i.e., a bimodal f/f_0. A separate f/f_0 of about 1.5 was fitted for all oligomers from approximately 1.3 to 20 S at every time point during the kinetics. The remaining signal includes a majority of monomer at any time point, resulting in a slightly higher f/f_0 of about 1.6. The ratio f/f_0 is a parameter of SV analysis that is usually fitted globally for each data set, with the drawback that it is dominated by the *s*-value species with the highest fraction of signal. Since our $c(s)$ distributions present a baseline separation between the monomer peak (~0.67 S) and oligomers (starting from ~1.7 S) for most samples, it was possible to apply the $c(s)$ model with prior knowledge, i.e., a bimodal f/f_0. The use of this model enables separate determination of shape factors for the monomer fraction and oligomer fraction of a sample, enhancing the overall fitting quality and mass calculation. The group of oligomers is composed of at least three distinct populations. We assign two single peaks at ~1.7 and ~2.8 S to two distinct LMWOs. The third population is assigned to a group of HMWOs represented as a broader distribution between 4 and 20 S. Since HMWOs make up the majority of all oligomers, their calculated masses based on the f/f_0 value of 1.5 are expected to be more accurate than the masses of low-abundance LMWOs described by the identical f/f_0 value. In Fig. 2 *D*, the weight average *s*-values for monomer and the three groups of oligomers are shown as the result of the integration of the $c(s)$ distributions. The comparison shows a persistent *s*-value for monomer as well as the two LMWO species with little deviation during the aggregation process. In contrast to that, the broader distribution of HMWOs exhibits a steady increase of the weighted average *s*-value from 1 to 19 h. This increase is observed to stagnate as soon as the system enters the rapid growth phase of amyloid fibrils. It should be noted that the signal for determination of the weighted average *s*-value for the last two time points is very small due to the high number of large aggregates at this stage of aggregation. This change in weight average *s*-value can be due to a change in mass or shape.

After ~18 h, at the end of the lag phase, the system reaches the rapid growth phase, which is accompanied by a strong consumption of monomers, pointing to the presence of growth-competent nuclei. At this stage, there is a steadily increasing loss of total Aβ42 concentration, indicating the presence of large aggregates. As a result, the proportion of monomers and oligomers decreases.

Noncovalent fluorescence staining of intermediate Aβ42 species

An assignment was established between aggregate species detected by SV experiments at certain time points during fibril formation and the corresponding ThT fluorescence signal. Large aggregates sedimenting during acceleration of the AUC are ThT positive, and at least part of the ThT-fluorescence signal obtained during the lag phase of amyloid fibril formation is caused by ThT-stainable oligomers. To further investigate the structural properties of oligomeric species formed before the amyloid fibrils, we tested a set of fluorescence dyes with a variety of different scaffolds and net charges for their capability in binding to oligomers during SV experiments. Although the SV analysis allows a qualitative as well as quantitative characterization of Aβ42 assemblies in solution, it suffers from the high fraction of monomeric species for most of the time points, which limits the precision for oligomer detection. Utilizing fluorescence probes increases the resolution of the species of interest since the chosen extrinsic dyes stain only Aβ42 oligomers but not monomers.

We decided to use an incubation time (6 h) with a sufficient number of oligomers but well before amyloid fibril formation to avoid the presence of competing binding sites for the fluorescence dyes. As the FDS of the AUC uses a 488-nm laser for excitation, the selection of fluorescence dyes is limited, and ThT has to be excluded. With an extrinsic, noncovalently binding dye, which can be added shortly before SV analysis and is therefore absent during incubation, a possible impact on the aggregation behavior is minimized. Assuming that a suitable dye is presenting an increase in fluorescence emission upon binding to a specific molecular assembly, we filter the molecular mixture of different species for only the positive staining results. These stained species can be observed during their sedimentation process in the presence of fluorescence dyes. Unbound dyes can be expected not to sediment due to their low molecular mass. We believe that this method can provide more detailed information on the specificity of protein binding dyes for particular oligomeric species than aggregation kinetics or histological staining reactions, which lack information on oligomer size. Our selection of suitable dyes included SO, hFTAA, SG, DCVJ, and YOYO (Table S1).

Fig. 3, *A–F*, show the overlays of the $c(s)$ distributions from one absorbance measurement (black) and separate fluorescence measurements with each dye. The corresponding raw data are shown in Fig. S5. It illustrates that all fluorescence dyes have the capability to stain the group of HMWOs between 4 and 20 S but are limited in binding to the smaller LMWOs. The weighted average s-value for all distributions is similar except for YOYO (Table S2), indicating either no specific staining preferences of the fluorescence dyes or no difference in binding sites among the group of HMWOs. YOYO seemed to stain preferentially larger s-value species compared with other dyes. Further experiments showed an aggregation-promoting effect of YOYO. This explanation arises from the dependence of the observed enhancement of aggregation on the duration of exposure of the Aβ42 peptide to YOYO (Fig. S6, *A* and *B*). The incubation of the Aβ42 peptide solution in the presence of the fluorescence dye is limited to the time required to establish the vacuum and equilibrate the temperature before starting SV experiments (about 1 h).

Looking at the single distributions (Fig. 3 *A–E*) overlaid with an absorbance measurement, it is evident that all fluorescence dyes report the smallest species at an s-value between 2 and 3 S. This species was already identified as a penta-/hexameric LMWO in the time series of absorbance measurements at ~2.8 S (Fig. 2 *D*). No fluorescence dye reports a clear peak for the smallest, otherwise detectable oligomer, i.e., the tri/tetrameric LMWO at ~1.7 S from absorbance measurements or the monomer at ~0.6 S. The s-value distributions show that all selected dyes generally enhance the analysis of intermediate species by ignoring the large monomer fraction of the peptide solution. The selected dyes differ in structure and overall charge, presenting different surfaces for the detection of structural

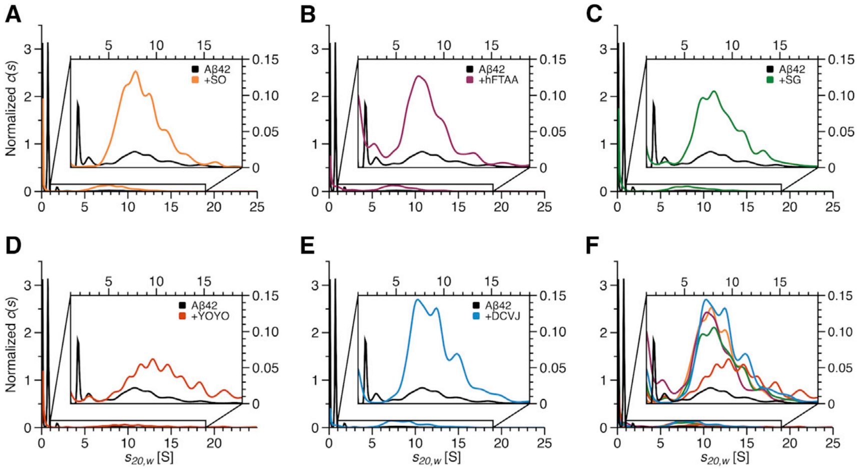

FIGURE 3 Fluorescence staining of Aβ42 oligomers. (*A*)–(*E*) show the $c(s)$ distributions based on fluorescence measurements for 40 μM Aβ42 in the presence of different fluorescent dyes, SO (orange), hFTAA (dark red), SG (green), YOYO (red), and DCVJ (cyan). In each case, the $c(s)$ distribution of a separate absorbance measurement (*black*) without dye was added as a reference. (*F*) shows the superposition of all curves. All samples were measured in 20 mM sodium phosphate buffer (pH 7.4) with 25 mM NaCl (2% dimethyl sulfoxide for samples with fluorescence dyes) at 20°C and 60,000 Rpm. The incubation time before analysis was 6 h. Magnifications visualize oligomeric species from 1 to 19 S. The $c(s)$ distributions were normalized to one according to the area under the curve for the complete s-value range from 0 to maximum s-value. To see this figure in color, go online.

differences among oligomeric species. The high similarity of the reported s-value distributions for all fluorescence dyes supports common structural features of oligomeric species. This is particularly interesting for the group of HMWOs, as the labeling with either SO, SG, hFTAA, or DCVJ shows no significant differences in terms of affinity for specific s-value species within this population.

Comparison of intermediates for different Aβ variants

After having determined the evolution of Aβ42 aggregate size distributions over time, we wanted to know how the oligomer size distributions of closely related Aβ peptides behave. For comparison, we selected the slightly shorter Aβ40 and Aβ42 with the mono-oxidized methionine in position 35, Aβ42 M35^{ox}. For Aβ40, as well as for Aβ42 M35^{ox}, it is known that they aggregate less vigorously than Aβ42 (32,33). Fig. 4, *A–D*, shows $c(s)$ distributions for the two variants compared with the Aβ42 peptide. Next to the monomeric peak at ~0.67 S for all variants, the smallest oligomer was detected at ~1.7 S consistently for all variants. Aβ40, as well as Aβ42 M35^{ox}, show a lower amount of HMWOs after the same incubation time at the same concentration and solvent conditions as Aβ42. Additionally, the weighted average s-value for the HMWOs is smaller, presented by the shift of $c(s)$ distribution toward smaller s-values. The overall fractions of intermediate species are only 12.4% and 9.6% for Aβ42 M35^{ox} and Aβ40, respectively, compared with 27.5% for Aβ42. The formation of LMWOs, especially the tri/tetrameric species, is not affected by the difference between the variants and Aβ42. Since the amyloid formation of both variants was so slow under the chosen conditions, we had to refrain from analyzing further time points.

DISCUSSION

Since 2004, when Klein et al. published that small Aβ assemblies, instead of fibrils, are the proximate neurotoxin in AD, numerous studies have been undertaken to identify and characterize these small assemblies (34). Especially under *in vitro* conditions, oligomeric assemblies are undeniable reaction intermediates present during the aggregation process of Aβ. A common classification of oligomer species is based on their contribution to the amyloid-formation process, with those oligomers that react further to form amyloid fibrils referred to as on-pathway oligomers and those oligomers that result from side reactions referred to as off-pathway oligomers (35,36). But this distinction in on- and off-pathway species can get difficult if only a fraction of an oligomeric species converts into an amyloid structure, while the residual part of this species dissociates back into monomers. According to (37), the classification of an oligomeric species should not depend on the size of the fraction that converts to amyloid but on the amount of amyloid that emerges from that oligomeric species. Therefore, an oligomeric species is on pathway if it promotes nucleation leading to amyloid growth even if its bulk dissolves back into monomers. Any on-pathway assembly with a size equal to or larger than the nucleus will not be detectable because of its fast growth. In this study, the time course of Aβ42 oligomer formation was studied.

In parallel with ThT kinetics, the temporal evolution of the s-value distribution of oligomeric assemblies of the Aβ42 peptide was analyzed. The increase in ThT fluorescence, complemented by CD and AFM results, confirming the β-sheet structure and fibril-like shape of the final products, demonstrate that the presented in vitro aggregation system models the amyloid-formation process. SV experiments as a technique to study oligomeric species proved to be especially suited. Since the aggregation process seems to be frozen during AUC, it was possible to successfully fit the experimental SV data with a $c(s)$ model for noninteracting species (25), as supported by the low values of the root-mean-square deviation of ≤1% of the total signal. The accuracy of the SV analysis was demonstrated by the threefold reproduction of results for independently prepared samples for different time points of the Aβ42 aggregation process. Since primary nucleation in this scenario has the

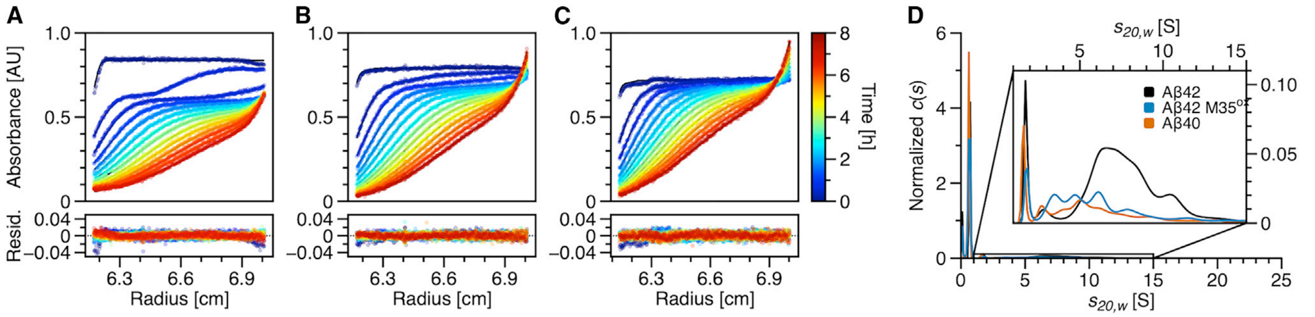

FIGURE 4 Comparison of Aβ variants in SV analysis. Raw data with fitted Lamm equation solutions are shown in (*A*), (*B*), and (*C*) for 40 μM Aβ42, Aβ42 M35^{ox}, and Aβ40, respectively, in 20 mM sodium phosphate buffer (pH 7.4) with 25 mM NaCl measured at 20°C and 60,000 Rpm. The resulting $c(s)$ distributions after 1 h incubation are shown in (*D*). (Weighted average s-values for HMWOs can be found in Table S2.) The $c(s)$ distributions were normalized to one according to the area under the curve for the complete s-value range from 0 to maximum s-value. To see this figure in color, go online.

highest energy barrier to the formation of Aβ amyloids, we expected that this process would be largely absent during measurement. Concerns about the effects of increased hydrostatic pressure and shear forces associated with high-speed centrifugation techniques were addressed using reduced speed experiments (Fig. S7).

In Fig. 5, a schematic view of the aggregation process as derived from this study is shown. The aggregation mechanism starts on the left from a supersaturated monomer solution, which forms a growth-competent nucleus either by chance or via nucleation sites (38), e.g., within or on the surface of HMWOs. The final product on the right is the amyloid fibril structure.

The unstructured monomer is in rapid equilibrium with LMWOs. The LMWOs of this study are subnucleus-sized oligomers, i.e., small assemblies with sizes below the critical nucleus. They have s-values of $\sim$1.7 and $\sim$ 2.8 S with hydrodynamic radii of 2 and 5 nm, respectively, and can be related to species already described in literature (15,16,32,39,40) ranging from trimers to hexamers of Aβ42. Since their proportion in the total sample is very small, the application of the averaged shape factor for the conversion into molar masses is the most error prone for these species. However, this uncertainty is well reflected by the inclusion or exclusion of a monomer unit. Their s-value is constant over the complete incubation period, but upon entry into the growth phase, their amount is the first to decrease after the monomer concentration starts to drop (Fig. S2).

Aside from LMWOs, HMWOs are also present from the first time point onward. They are separated in $c(s)$ curves from the LMWOs by a minimum located between 3 and 4 S. Based on their specific shape factor $f/f_0 = 1.5$ and the weighted average s-value, the molar mass and thus the number of monomeric units could be calculated for the group of HMWOs. After 1 h incubation, the observed s-value distribution between 4 and 20 S corresponds to about 35–47 monomeric units per assembly. Oligomers of a similar size (30–50 monomers) with a hydrodynamic radius of 7 nm had been characterized by small-angle neutron scattering (41). The average number of monomer units of HMWOs doubles to 78–89 monomer units by the end of the lag phase. No evidence was found for a change in shape as a cause for the increase in the averaged s-value of the HMWOs. The result that virtually no species with s-values between 20 and 208 S (the detection limit at 60,000 Rpm) were detected during amyloid formation suggests that nucleation occurs in the s-value range up to 20 S. Once formed, outgrowth must be a rapid process leading directly to structures with s-values above 208 S, which are almost immediately removed from solution by sedimentation during the acceleration phase. This in turn means that the nucleus formed under these conditions is extremely competent in monomer recruitment. Their fraction decreases last when the system enters the rapid growth phase, where amyloid fibril elongation and secondary nucleation are the dominant processes. A possible explanation for the gradual growth is the energetic favor of larger assemblies due to a decrease in surface energy in analogy to a process known from micelle structures or protein clusters, termed Ostwald ripening. Similar observations were made with lysozyme protein clusters during incubation for the purpose of crystal formation (42,43). Furthermore, the increase in size stops as soon as crystallization starts, again resembling what we observe for Aβ42 aggregation. Alternatively, cluster coalescence could be the reason for the increase in size. Despite the high local Aβ concentration and the early presence of the β-strand structure (44), the conformational change or consolidation required to form a growth-competent nucleus still takes several hours. Nevertheless, the nucleation mediated by HMWOs is presumably more probable and outperforms the spontaneous primary nucleation free in solution (dashed line connecting monomers and nucleus) at protein concentrations above a critical value.

The identified subnucleus-sized LMWOs exist at the same time as a heterogeneous population of HMWOs, which appear to foster nucleation by providing high local Aβ concentrations. HMWOs act as on-pathway species when they promote the formation of nuclei. However, after entering the growth phase, remaining HMWOs are dissolved in terms of off-pathway species as a result of monomer depletion. LMWOs are less stable and disappear before HMWOs (Fig. S2). The role of HMWOs constituting potential nucleation sites was suggested in our recent review (38).

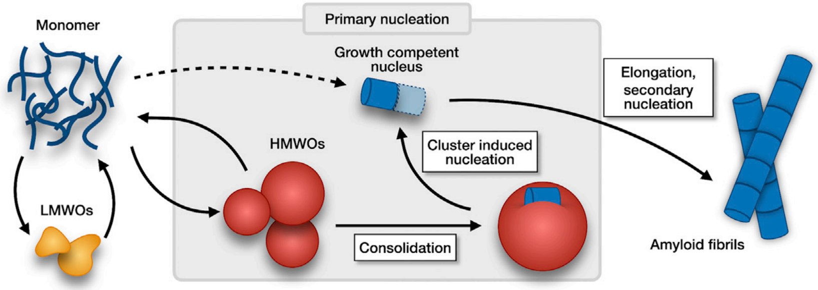

FIGURE 5 Aggregation mechanism of Aβ42 in the presence of subnucleus-sized LMWOs and nucleation-mediating HMWOs. Monomers are in equilibrium with LMWOs (3–6 monomeric units) and HMWOs (20–160 monomeric units). Either consolidation of HMWOs or formation of an elongation competent LMWO leads to the formation of a nucleus competent in monomer recruitment that initiates amyloid formation. LMWOs and then HMWOs dissolve upon aggravated monomer consumption through fibril elongation and secondary nucleation. To see this figure in color, go online.

This hypothesis is further supported by the results that Aβ40 as well as Aβ42 M35^{ox}, which have a much longer lag phase than Aβ42 under identical solvent and concentration conditions, form less and smaller HMWOs in solution than Aβ42, while the size and amount of subnucleus-sized LMWOs are comparable. Further, this observation is in line with the findings for the Icelandic mutant (A2T) of Aβ42, which forms in vitro only 50% or fewer oligomers of high molecular weight (>50 kDa corresponding to >11 monomeric units) compared with *wt* protein at the same concentration (45) and significantly reduces the risk for Alzheimer's dementia. The studied variants of the Aβ peptide possess lower hydrophobicity than Aβ42, thus pointing to the hydrophobic effect as a driving force for HMWO formation. Therefore, we conclude that cluster-induced nucleation contributes as another mechanism to amyloid formation in addition to primary and secondary nucleation (46). This raised the question of whether there is a critical cluster concentration above which the concentration of free monomers remains constant while the total concentration of Aβ42 increases. A concentration series for Aβ42 from 5 to 40 μM peptide gave a linear dependence of free monomer to total Aβ42 concentration (Fig. S3). This indicates that a potential critical cluster or oligomer concentration has to be higher than 40 μM.

Since the HMWO species appeared to be not a single but multiple species within the determined *s*-value distributions, the question arose of whether the heterogeneity would manifest itself in deviating structural properties. Since a physical separation of the different types of HMWOs proved to be unfeasible, a method combining the fractionating properties of AUC and fluorescence staining was chosen. The finding of no differences in stainability is consistent with a uniform micelle or cluster-like structure of HMWOs regardless of size (38). Monomers and the 1.7 S species could not be stained, probably because their small size does not provide binding sites for the dyes. A possible explanation for the aggregation-enhancing property of YOYO could be its high positive charge, which was exclusive in the group of tested fluorescence dyes. Electrostatic attraction forces between dye and peptide could lead to the formation of particles that tend to aggregate as charge repulsion is reduced. The biological on-line tracer-type SV experiments clearly demonstrated that SO can effectively stain oligomeric species of Aβ42. This is in contrast to statements that SO as an amyloid dye does not stain oligomers (21,22). How much the resolution for oligomeric species in SV experiments is improved by the use of a fluorescent dye depends strongly on the quantum yield and the difference between bound and unbound states of the fluorescent dye.

In addition to monomer and fibril, we have identified several intermediate structures that play different roles in the process of amyloid formation and may serve as potential targets for future therapeutic approaches. Aβ42 HMWOs of similar size to those in our study were detected immunologically at physiological Aβ42 concentrations (1–20 nM), with a 48-mer being the prominent species (47). In the same study, no comparable oligomerization was observed for Aβ40, in agreement with the lower concentrations of HMWOs determined for Aβ40 in our work. LMWOs are less stable than HMWOs and have a higher diffusivity. The toxic capacity of small diffusible species has already been presented (6). Possibly, the amyloid-formation process poses a rescue of the organism from the highly diffusible small oligomeric species. The neuronal damage will probably happen before amyloid deposits are formed due to such ADDLs. Therefore, LMWOs represent potential targets to address the elimination of diffusible species with potential neurotoxicity.

SUPPORTING MATERIAL

Supporting material can be found online at https://doi.org/10.1016/j.bpj.2022.12.020.

AUTHOR CONTRIBUTIONS

T.P., T.Z., and L.N.-S. designed the research. T.P. and R.S.-H. performed research and analyzed the data. T.P., L.N.-S., and D.W. wrote the article.

ACKNOWLEDGMENTS

Heinrich-Heine-University Düsseldorf, Germany, supported the project according to the agreement no. 42025376 (Technology and Medicine – Multimodal imaging to explore the in vivo behavior of polymeric biomaterials). We would like to sincerely thank the reviewers for their effort in reviewing our manuscript.

DECLARATION OF INTERESTS

D.W. is founder and shareholder of the companies Priavoid and Attyloid and is a member of their supervisory boards. This had no influence on the interpretation of the data.

REFERENCES

1. Cline, E. N., M. A. Bicca, …, W. L. Klein. 2018. The amyloid-beta oligomer hypothesis: beginning of the third decade. *J. Alzheimers Dis.* 64:S567–S610.
2. van Groen, T., S. Schemmert, …, D. Willbold. 2017. The Aβ oligomer eliminating D-enantiomeric peptide RD2 improves cognition without changing plaque pathology. *Sci. Rep.* 7:16275.
3. Schemmert, S., E. Schartmann, …, D. Willbold. 2019. Aβ oligomer elimination restores cognition in transgenic Alzheimer's mice with full-blown pathology. *Mol. Neurobiol.* 56:2211–2223.
4. Sanders, O. D., L. Rajagopal, and J. A. Rajagopal. 2022. The oxidatively damaged DNA and amyloid-β oligomer hypothesis of Alzheimer's disease. *Free Radic. Biol. Med.* 179:403–412.
5. Nagel-Steger, L., M. C. Owen, and B. Strodel. 2016. An account of amyloid oligomers: facts and figures obtained from experiments and simulations. *Chembiochem.* 17:657–676.

6. Wen, J., F. Fang, …, T. Hung. 2018. Amyloid β-derived diffusible ligands (ADDLs) induce abnormal autophagy associated with Aβ aggregation degree. *J. Mol. Neurosci.* 64:162–174.

7. Barghorn, S., V. Nimmrich, …, H. Hillen. 2005. Globular amyloid β-peptide1- 42 oligomer- a homogenous and stable neuropathological protein in Alzheimer's disease. *J. Neurochem.* 95:834–847.

8. Benilova, I., E. Karran, and B. De Strooper. 2012. The toxic Abeta oligomer and Alzheimer's disease: an emperor in need of clothes. *Nat. Neurosci.* 15:349–357.

9. Gremer, L., D. Schölzel, …, G. F. Schröder. 2017. Fibril structure of amyloid-beta(1-42) by cryo-electron microscopy. *Science.* 358:116–119.

10. Fitzpatrick, A. W., and H. R. Saibil. 2019. Cryo-EM of amyloid fibrils and cellular aggregates. *Curr. Opin. Struct. Biol.* 58:34–42.

11. König, A. S., N. S. Rösener, …, H. Heise. 2021. Structural details of amyloid βoligomers in complex with human prion protein as revealed by solid-state MAS NMR spectroscopy. *J. Biol. Chem.* 296

12. Sear, R. P. 2006. On the interpretation of quantitative experimental data on nucleation rates using classical nucleation theory. *J. Phys. Chem. B.* 110:21944–21949.

13. Linse, S. 2019. Mechanism of amyloid protein aggregation and the role of inhibitors. *Pure Appl. Chem.* 91:211–229.

14. Jana, M. K., R. Cappai, …, G. D. Ciccotosto. 2016. Membrane-bound tetramer and trimer Aβ oligomeric species correlate with toxicity towards cultured neurons. *J. Neurochem.* 136:594–608.

15. Wolff, M., D. Unuchek, …, L. Nagel-Steger. 2015. Amyloid β oligomeric species present in the lag phase of amyloid formation. *PLoS One.* 10:e0127865.

16. Wolff, M., B. Zhang-Haagen, …, L. Nagel-Steger. 2017. Abeta42 pentamers/hexamers are the smallest detectable oligomers in solution. *Sci. Rep.* 7:2493.

17. Dam, J., C. A. Velikovsky, …, P. Schuck. 2005. Sedimentation velocity analysis of heterogeneous protein-protein interactions: lamm equation modeling and sedimentation coefficient distributions c (s). *Biophys. J.* 89:619–634.

18. Kroe, R. R., and T. M. Laue. 2009. NUTS and BOLTS: applications of fluorescence-detected sedimentation. *Anal. Biochem.* 390:1–13.

19. Nagarajan, S., and L. J. Lapidus. 2017. Fluorescent probe DCVJ shows high sensitivity for characterization of amyloid beta-peptide early in the lag phase. *Chembiochem.* 18:2205–2211.

20. Mok, Y. F., T. M. Ryan, …, M. D. W. Griffin. 2011. Sedimentation velocity analysis of amyloid oligomers and fibrils using fluorescence detection. *Methods.* 54:67–75.

21. Mora, A. K., and S. Nath. 2020. SYPRO Orange–a new gold standard amyloid probe. *J. Mater. Chem. B.* 8:7894–7898.

22. Wong, A. G., and D. P. Raleigh. 2016. The dye SYPRO orange binds to amylin amyloid fibrils but not pre-fibrillar intermediates. *Protein Sci.* 25:1834–1840.

23. Klingstedt, T., A. Åslund, …, K. P. R. Nilsson. 2011. Synthesis of a library of oligothiophenes and their utilization as fluorescent ligands for spectral assignment of protein aggregates. *Org. Biomol. Chem.* 9:8356–8370.

24. Lindberg, D. J., and E. K. Esbjörner. 2016. Detection of amyloid-beta fibrils using the DNA-intercalating dye YOYO-1: binding mode and fibril formation kinetics. *Biochem. Biophys. Res. Commun.* 469:313–318.

25. Schuck, P. 2000. Size-distribution analysis of macromolecules by sedimentation velocity ultracentrifugation and lamm equation modeling. *Biophys. J.* 78:1606–1619.

26. Adalsteinsson, D., and P. Schultz. 2020. The DataGraph Manual. Visual Data Tools, 4.6 edition.

27. Laue, T., B. Shah, …, S. Pelletier. 1992. Sednterp. *In* Analytical Ultracentrifugation in Biochemistry and Polymer Science. S. Harding and A. Rowe, eds Royal Society of Chemistry, Cambridge, pp. 90–125.

28. Biancalana, M., and S. Koide. 2010. Molecular mechanism of Thioflavin-T binding to amyloid fibrils. *Biochim. Biophys. Acta.* 1804:1405–1412.

29. Sabaté, R., and J. Estelrich. 2005. Evidence of the existence of micelles in the fibrillogenesis of beta-amyloid peptide. *J. Phys. Chem. B.* 109:11027–11032.

30. Lamm, O. 1929. Die differentialgleichung der Ultrazentrifugierung. *Ark. Mat. Astr. Fys.* 21B:1–4.

31. Hellstrand, E., B. Boland, …, S. Linse. 2010. Amyloid β-protein aggregation produces highly reproducible kinetic data and occurs by a two-phase process. *ACS Chem. Neurosci.* 1:13–18.

32. Nirmalraj, P. N., J. List, …, M. Mayer. 2020. Complete aggregation pathway of amyloid β(1-40) and (1-42) resolved on an atomically clean interface. *Sci. Adv.* 6:eaaz6014.

33. Friedemann, M., E. Helk, …, V. Tõugu. 2015. Effect of methionine-35 oxidation on the aggregation of amyloid-β peptide. *Biochem. Biophys. Rep.* 3:94–99.

34. Klein, W. L., W. B. Stine, Jr., and D. B. Teplow. 2004. Small assemblies of unmodified amyloid β-protein are the proximate neurotoxin in Alzheimer's disease. *Neurobiol. Aging.* 25:569–580.

35. Ehrnhoefer, D. E., J. Bieschke, …, E. E. Wanker. 2008. EGCG redirects amyloidogenic polypeptides into unstructured, off-pathway oligomers. *Nat. Struct. Mol. Biol.* 15:558–566.

36. Baskakov, I. V., G. Legname, …, F. E. Cohen. 2002. Pathway complexity of prion protein assembly into amyloid. *J. Biol. Chem.* 277:21140–21148.

37. Dear, A. J., G. Meisl, …, T. P. J. Knowles. 2020. Identification of on- and off-pathway oligomers in amyloid fibril formation. *Chem. Sci.* 11:6236–6247.

38. Willbold, D., B. Strodel, …, H. Heise. 2021. Amyloid-type protein aggregation and prion-like properties of amyloids. *Chem. Rev.* 121:8285–8307. https://doi.org/10.1021/acs.chemrev.1c00196.

39. Bitan, G., M. D. Kirkitadze, …, D. B. Teplow. 2003. Amyloid β-protein (Aβ) assembly: aβ40 and Aβ42 oligomerize through distinct pathways. *Proc. Natl. Acad. Sci. USA.* 100:330–335.

40. Bernstein, S. L., N. F. Dupuis, …, M. T. Bowers. 2009. Amyloid-β protein oligomerization and the importance of tetramers and dodecamers in the aetiology of Alzheimer's disease. *Nat. Chem.* 1:326–331.

41. Yong, W., A. Lomakin, …, G. B. Benedek. 2002. Structure determination of micelle-like intermediates in amyloid β-protein fibril assembly by using small angle neutron scattering. *Proc. Natl. Acad. Sci. USA.* 99:150–154.

42. Streets, A. M., and S. R. Quake. 2010. Ostwald ripening of clusters during protein crystallization. *Phys. Rev. Lett.* 104:178102. https://doi.org/10.1103/PhysRevLett.104.178102.

43. Li, Y., V. Lubchenko, …, P. G. Vekilov. 2012. Ostwald-like ripening of the anomalous mesoscopic clusters in protein solutions. *J. Phys. Chem. B.* 116:10657–10664. https://doi.org/10.1021/jp303316s.

44. Brener, O., T. Dunkelmann, …, D. Willbold. 2015. QIAD assay for quantitating a compound's efficacy in elimination of toxic Aβ oligomers. *Sci. Rep.* 5:13222–13313.

45. Limegrover, C. S., H. LeVine, III, …, S. M. Catalano. 2021. Alzheimer's protection effect of A673T mutation may be driven by lower Aβ oligomer binding affinity. *J. Neurochem.* 157:1316–1330.

46. Cohen, S. I. A., S. Linse, …, T. P. J. Knowles. 2013. Proliferation of amyloid-β42 aggregates occurs through a secondary nucleation mechanism. *Proc. Natl. Acad. Sci. USA.* 110:9758–9763.

47. LeVine, H., III 2004. Alzheimer's β-peptide oligomer formation at physiologic concentrations. *Anal. Biochem.* 335:81–90.

Biophysical Journal, Volume *122*

Supplemental information

Differentiation of subnucleus-sized oligomers and nucleation-competent assemblies of the Aβ peptide

Thomas Pauly, Tao Zhang, Rebecca Sternke-Hoffmann, Luitgard Nagel-Steger, and Dieter Willbold

Article

Differentiation of sub-nucleus sized oligomers and nucleation competent assemblies of the Aβ peptide

Thomas Pauly[1,2], Tao Zhang[1,2,3], Rebecca Sternke-Hoffmann[1,4], Luitgard Nagel-Steger[1,2,*], and Dieter Willbold[1,2]

[1]Institut für Physikalische Biologie, Heinrich-Heine-Universität Düsseldorf, 40225 Düsseldorf, Germany
[2]Institute of Biological Information Processing (IBI-7: Structural Biochemistry), Research Center Jülich, 52425 Jülich, Germany
[3]Present address: Fujian Key Laboratory of Translational Research in Cancer and Neurodegenerative Diseases, Institute for Translational Medicine, School of Basic Medical Sciences, Fujian Medical University, Fuzhou, Fujian 350122, China.
[4]Present address: Department of Biology and Chemistry, OFLC/108, Paul Scherrer Institut, Forschungsstrasse 111, 5232 Villingen, Switzerland
[*]Correspondence: luitgard.nagel-steger@hhu.de

SUPPLEMENTARY MATERIAL

Material and methods

Analytical ultracentrifugation

Due to the applied centrifugal forces hydrostatic pressure is build up in the sample forming a gradient with a maximum at the bottom of the cell. Since changing pressure can affect macromolecular structure or chemical equilibria, it is helpful to calculate with 1 how high this pressure is under the selected experimental conditions.

$$P = \omega^2 \rho (r_0 h + h^2/2) \tag{1}$$

In 1 P is the hydrostatic pressure above atmospheric pressure, ω the angular velocity, ρ the density of the fluid column, r_0 the radial distance from the axis of rotation to the meniscus , and h the radial position in the cell: the liquid column height (1).

In order to test for the impact of these side effects of centrifugation either the solution column can be reduced or the speed can be lowered. A run with reduced speed was chosen resulting not only in reduced hydrostatic pressure but also in less shear forces and less pronounced solvent inhomogeneity. The difference in hydrostatic pressure at the bottom of the cell by changing the speed from 60 kprm (294 bar at 7.19 cm) und 50 kprm (202 bar at 7.18 cm) is about 90 bar.

ThT data

The obtained ThT fluorescence data were fitted by a sigmoidal function that allows to obtain the lag time of amyloid fibrils formation (2). The fitted parameters are the elongation rate constant (k), the half completion time ($t^{1/2}$), the lower fluorescence baseline (F_0), and the amplitude to reach the upper baseline (A).

$$F(t) = F_0 + \frac{A}{1 + \exp(-k(t - t^{1/2}))} \tag{2}$$

AFM imaging

Aβ42 in 20 mM Sodium phosphate buffer with 25 mM sodium chloride was incubated for 48 h before dilution from 40 to 20 µM and preparation of AFM samples. MICA surfaces were incubated for 20 minutes with 10 µL peptide solution, followed by washing steps with water and dried with nitrogen. Images were obtained using a NanoScope V (Bruker) atomic force microscope equipped with a silicon cantilever ScanAsyst-Air with a tip radius of 2-12 nm. Images were processed with Gwyddion 2.56 for presentation.

Fluorescence dyes

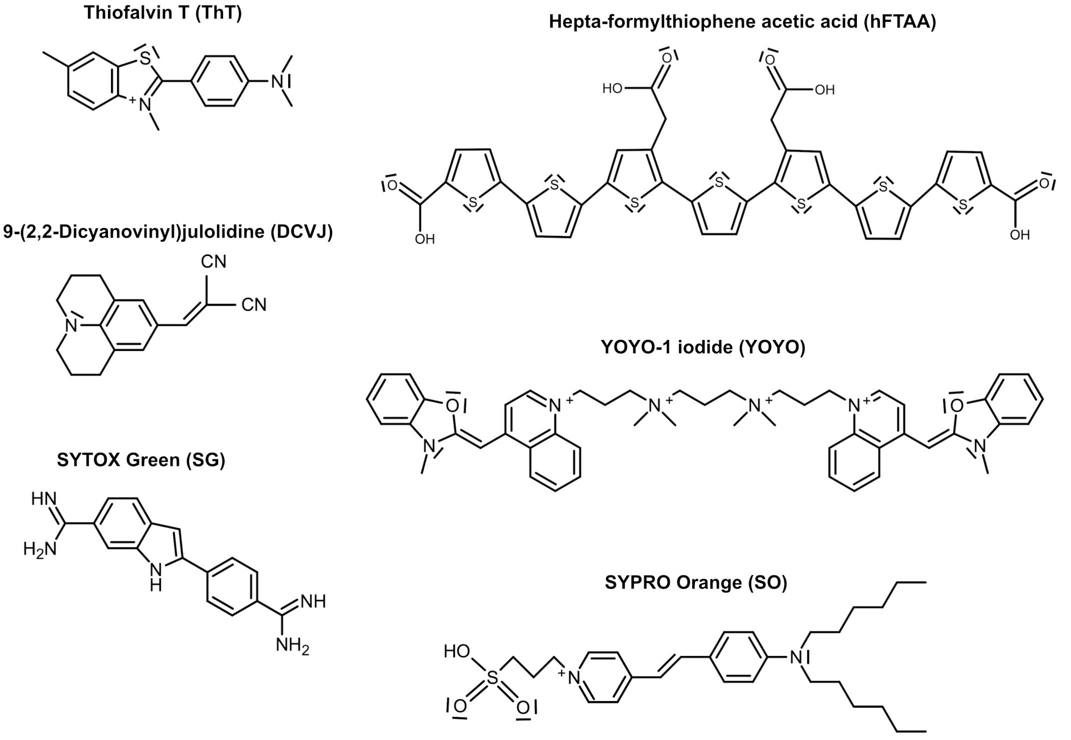

Figure S1: Molecular structures of fluorescent dyes used in this study.

Table S1: Fluorescent dyes with respective net charge and solvent used in this study

**Fluorescence dye**	**Source**	**Solvent**	**Charge** [$\times 1.602\times 10^{-19}$ C]
ThT	Sigma-Aldrich, Germany	Water	+ 1
DCVJ	Sigma-Aldrich, Germany	DMSO	neutral
SO	Sigma-Aldrich, Germany	DMSO	zwitterionic
hFTAA	Nilsson and Hammarström (3)	Water	− 4
YOYO	ThermoFisher, Germany	DMSO	+ 4

Results

Table S2: Weighted average *s*-value for oligomers from *c*(*s*) distributions.

Sample	Detection type	average *s*-value [S]
		**Oligomers from 4 to 20 S**
Aβ42	Absorbance	9.16
Aβ42 + SO	Fluorescence	8.63
Aβ42 + hFTAA	Fluorescence	8.66
Aβ42 + SG	Fluorescence	9.03
Aβ42 + YOYO	Fluorescence	11.02
Aβ42 + DCVJ	Fluorescence	9.24
		**Oligomers from 2 to 20 S**
Aβ42	Absorbance	8.65
Aβ42 M35^{ox}	Absorbance	6.92
Aβ40	Absorbance	7.12

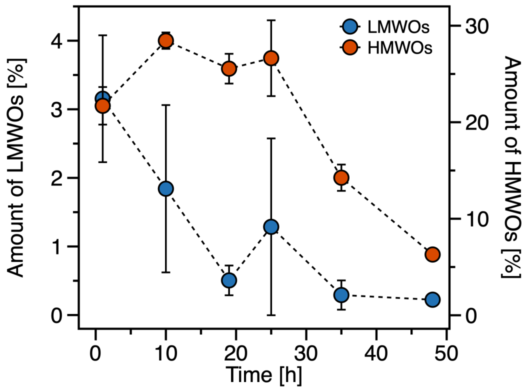

Figure S2: Amount of small oligomers and clusters of 40 µM Aβ42 for different incubation time points as the result of peak integration from 1.2 S to 4 S (blue dots) and 4 S to 20 S (red dots), respectively.

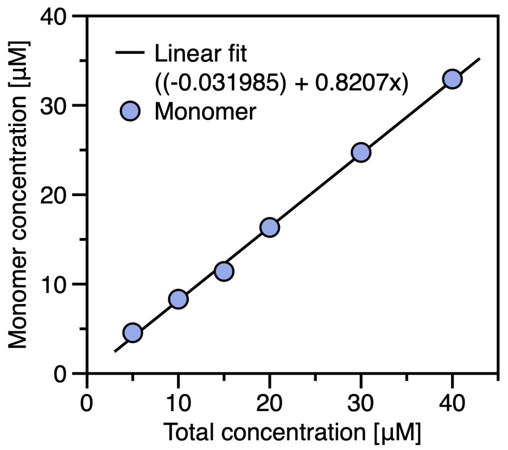

Figure S3: Integration of monomer peak from *c*(*s*) distributions obtained for a concentration series of Aβ42 without pre-incubation in 20 mM NaPi with 25 mM NaCl (pH 7.4). The monomer concentration dependence from total peptide concentration can be described by a linear relation between 5 and 40 µM.

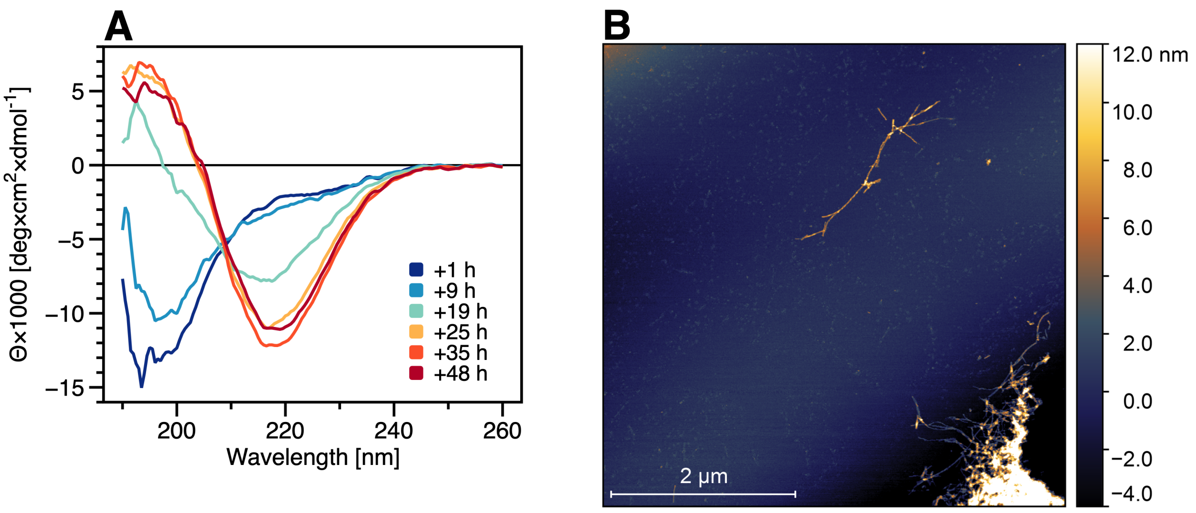

Figure S4: A shows CD spectra for 40 µM Aβ42 after different incubation times. B shows an AFM image of 20 µM Aβ42 after 48 h incubation. Samples were taken from kinetic experiments with 40 µM Aβ42 and diluted with 20 mM NaPi and 25 mM NaCl (pH 7.4).

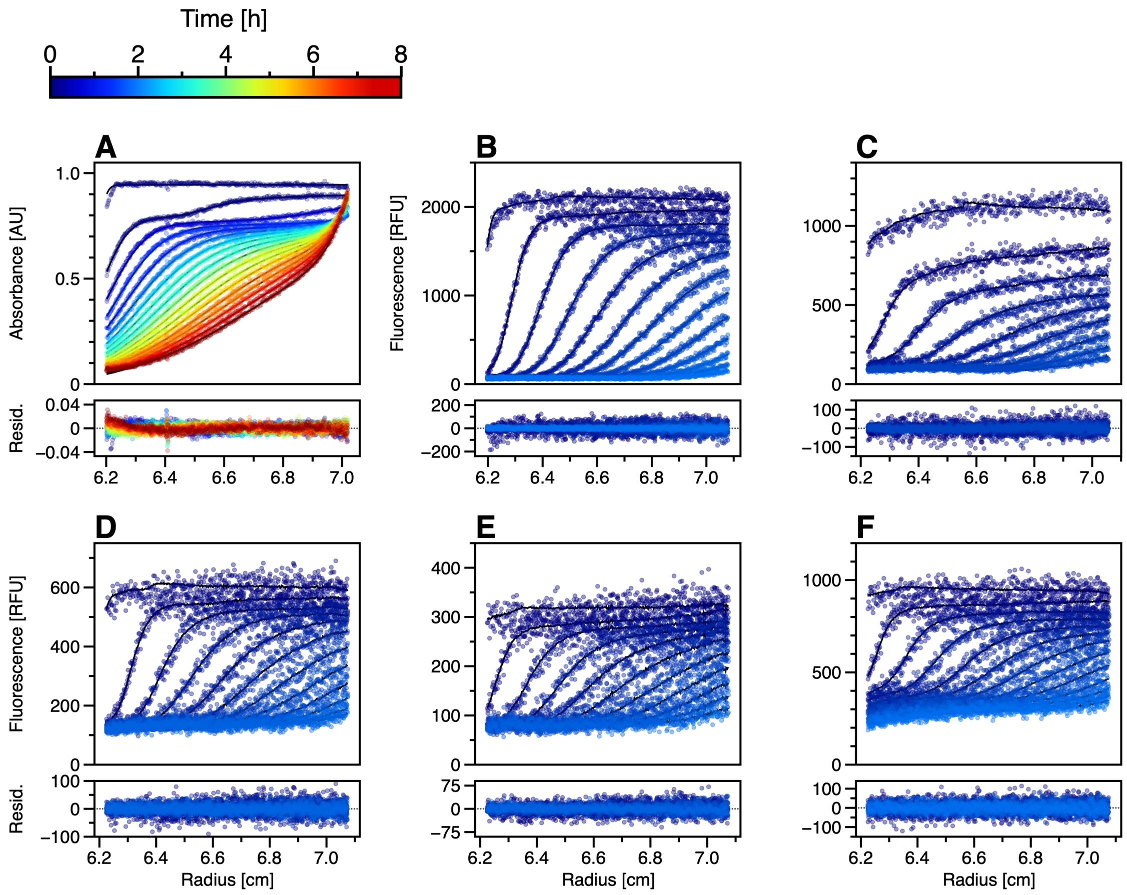

Figure S5: Sedimentation profiles of 40 µM Aβ42 after 6 h incubation measured with absorbance detection (A) and with fluorescence detection in the presence of different dyes: SO (B), YOYO (C), SYTOX (D), DCVJ (E) and hFTAA (F). The raw data is shown as points with fitted Lamm-equations solutions from the c(s) model implemented in Sedfit. For clarity, only every seventh scan is shown.

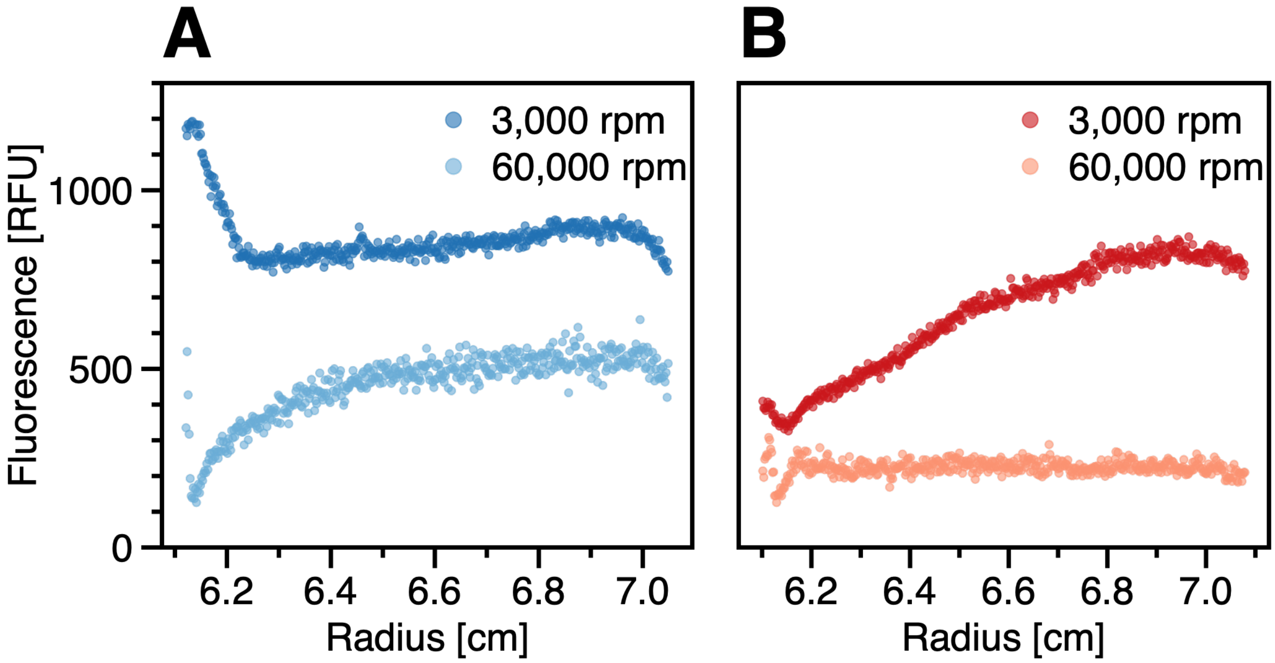

Figure S6: Loss of signal during incubation of 40 μM Aβ42 in the presence of YOYO. A single scan at 3,000 rpm and 60,000 rpm is shown for incubation of Aβ42 in the presence of YOYO for 1 h (A) and 6 h (B). The total incubation time of Aβ42 was 6 h for both samples.

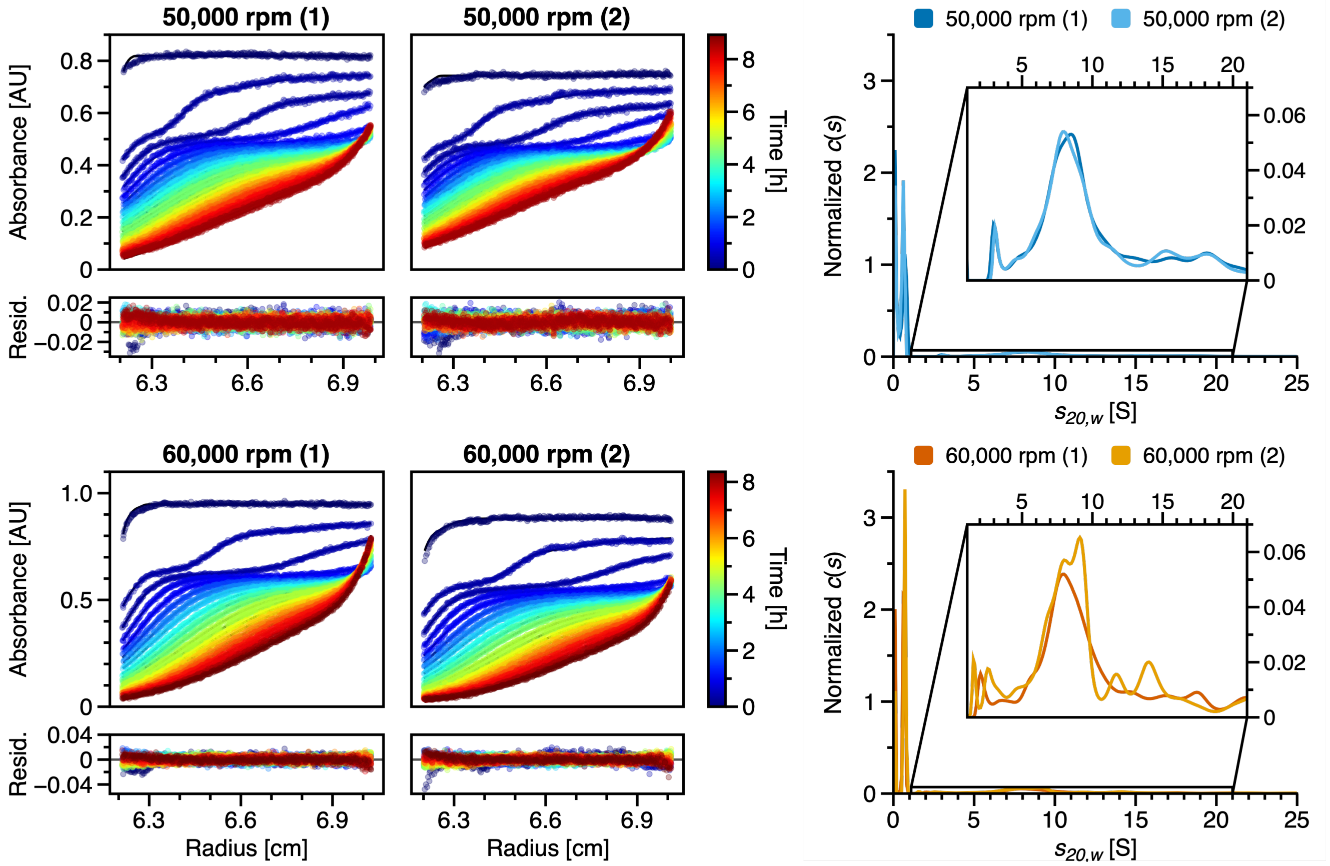

Figure S7: Sedimentation velocity analysis of 40 μM Aβ42 after 3 h incubation at 50,000 rpm and 60,000 rpm. Sedimentation profiles of duplicates show the raw data as points with fitted Lamm-equation solutions from the $c(s)$ model implemented in Sedfit. The resulting distribution of s-values is shown for duplicates of both speeds with magnification of oligomer distributions between 1 S and 21 S. Curves are normalized for the complete area under curve.

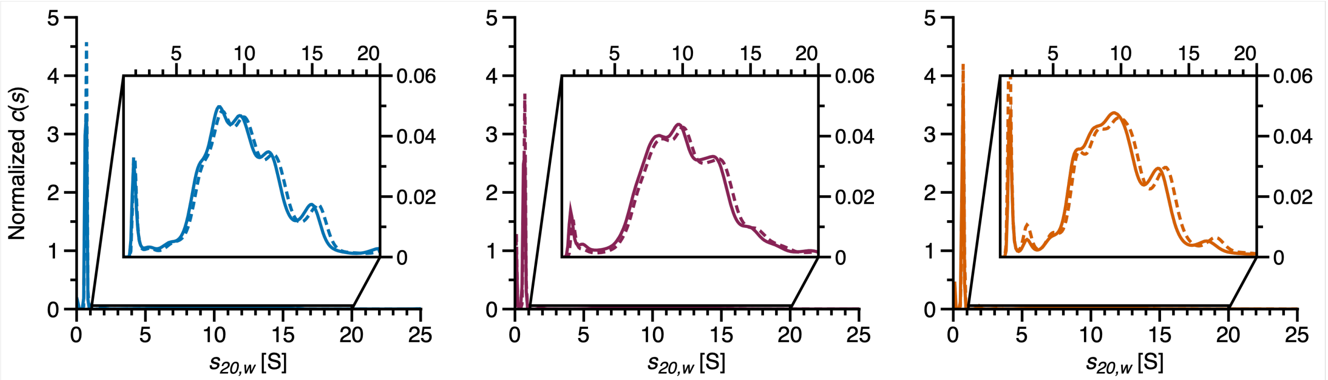

Figure S8: Comparison of sedimentation velocity analyses of 40 μM Aβ42 after 10 h incubation accounting for solvent compressibility. The data shown for triplicats from the main text (solid lines) were analyzed again using the $c(s)$ model with solvent compressibility (dashed lines) implemented in Sedfit.

REFERENCES

1. Molina-García, A., 1999. Hydrostatic pressure in ultracentrifugation. *In* Progress in Colloid and Polymer Science. Springer Verlag, volume 113, 57–61.

2. Hellstrand, E., B. Boland, D. M. Walsh, and S. Linse, 2010. Amyloid β-protein aggregation produces highly reproducible kinetic data and occurs by a two-phase process. *ACS chemical neuroscience* 1:13–18.

3. Klingstedt, T., A. Åslund, R. A. Simon, L. B. Johansson, J. J. Mason, S. Nyström, P. Hammarström, and K. P. R. Nilsson, 2011. Synthesis of a library of oligothiophenes and their utilization as fluorescent ligands for spectral assignment of protein aggregates. *Organic & biomolecular chemistry* 9:8356–8370.
